# Severe neonatal complications and long-term health-related quality of life in very preterm and/or very low birth weight survivors: evidence from the Dutch Project on preterm and small for gestational age cohort

**DOI:** 10.1007/s11136-026-04185-0

**Published:** 2026-05-03

**Authors:** Corneliu Bolbocean, Paula van Dommelen, Sylvia van der Pal

**Affiliations:** 1 Nuffield Department of Primary Care Health Sciences, University of Oxford, Radcliffe Observatory Quarter, Woodstock Road, Oxford, OX2 6GG UK; 2 Department of Child Health, Netherlands Organisation for Applied Scientific Research (TNO), Schipholweg 77-89, 2316 ZL Leiden, The Netherlands

**Keywords:** Preterm birth, HUI3, SF-6D, Bronchopulmonary dysplasia, Intraventricular hemorrhage, Necrotizing enterocolitis

## Abstract

**Background:**

Understanding the impact of severe neonatal complications such as Bronchopulmonary Dysplasia (BPD), Intraventricular Hemorrhage (IVH), or Necrotizing Enterocolitis (NEC) on adult health-related quality of life (HRQoL) beyond the effect of prematurity itself is significant for health economic evaluation and policy.

**Objective:**

Analyze the independent associations of BPD, IVH, NEC and multiple birth status with preference-based HRQoL utility scores in adulthood for survivors in the Dutch Project on Preterm and Small-for-gestational-age infants (POPS) a national cohort born in 1983.

**Methods:**

Exposures were documented neonatal BPD, severe IVH (grades 3–4), NEC, and multiple birth status. HRQoL data were available for n = 644 (19 y), n = 314 (28 y), and n = 370 (35 y). Using multivariable linear regression adjusted for confounders, we assessed the association between each exposure and HRQoL utility scores and optimal functioning. Analyses incorporated inverse probability weighting to adjust for potential attrition bias. We conducted comprehensive sensitivity analyses including best-case/worst-case imputation scenarios, comparison of IPW-weighted versus unweighted estimates, and post-hoc power calculations.

**Results:**

After adjustment for confounders, severe IVH (grade 3/4) was the only neonatal complication independently associated with significant and persistent decrements in overall preference-based HRQoL, with utility score reductions at 19 years (HUI3: *β* =  − 0.08, *p* = 0.05), 28 years (HUI3: *β* =  − 0.13, *p* = 0.01), and 35 years (SF-6D: *β* =  − 0.07, *p* = 0.04). These findings were robust to IPW adjustment for attrition (all |∆*β*| < 0.02) and fell within plausible bounds established by best-case/worst-case sensitivity analyses.

**Conclusion:**

Severe IVH was associated with significant and clinically meaningful utility decrements that persisted into the fourth decade of life.

## Introduction

Very premature (VP) births (< 32 weeks gestation) or with very low birth weight (VLBW) (< 1500 g), are associated with increased mortality risk [1] and significant adverse neurodevelopmental outcomes [2–4]. Advances in neonatal care have dramatically improved survival rates for these vulnerable infants [5, 6]. However, this success has led to a growing population of survivors who may face long-term health challenges and socio-economic difficulties extending into adulthood [7, 8].

Among preterm survivors, certain neonatal complications significantly increase the risk of adverse long-term outcomes, particularly for those born very preterm (< 32 weeks gestational age) [9]. Bronchopulmonary Dysplasia (BPD), a chronic lung disease; severe Intraventricular Hemorrhage (IVH), bleeding within the brain’s ventricles; and Necrotizing Enterocolitis (NEC), a severe intestinal disease, are among the most serious morbidities faced by preterm infants [10–17]. BPD is known predictor of neurodevelopmental impairment, respiratory problems [18–27] while NEC [28, 29] and IVH [30–32] impact the likelihood of chronic health issues persisting beyond childhood. Additionally, being born as part of a multiple birth (MB) is often associated with prematurity and low birth weight, potentially adding another layer of risk.

The long-term burden associated with these essentially unpredictable risk factors [33–40] substantial costs on healthcare systems and society [6, 41–46]. To effectively evaluate interventions and allocate resources, robust measures of health outcome are essential. Health-related quality of life (HRQoL) provides a comprehensive assessment of an individual’s well-being across physical, mental, and social domains [47, 48].

Preference-based health-related quality of life measures (HRQoL) such as Health Utilities Index Mark 3 (HUI3) [49, 50] and the Short Form 6 Dimensions (SF-6D) [51, 52] are standardized, multidimensional health state classifications that include preference or utility weights derived from representative population samples. Among these measures, the Health Utilities Index Mark 3 (HUI3) is a dominant measure due to its robust psychometric properties [53, 54] and it is the most widely used preference-based HRQoL measure in children [55–57]. These utility scores are critical inputs for cost-utility analyses, informing healthcare policy and funding decisions [58–60].

Although adult HRQoL after very preterm/very low birthweight birth has been studied [7, 61], evidence is scarce on how specific neonatal complications (BPD, severe IVH, NEC) or multiple birth affect preference-based utility in adulthood beyond prematurity itself. Condition-specific utility decrements are essential for valid economic evaluations (e.g., neuroprotective agents). Using average preterm utilities might mask heterogeneity and force non–evidence-based assumptions, weakening resource-allocation decisions and obscuring targets for intervention.

In this study, we aimed to investigate the impact of experiencing BPD, IVH, NEC, or being part of a multiple birth on preference-based HRQoL in adults born VP and/or VLBW. We utilized data from the Dutch Project on Preterm and Small-for-gestational-age infants (POPS), a unique, nationwide longitudinal cohort of individuals born VP and/or VLBW in 1983 and followed into adulthood [62]. Specifically, we sought to determine if these neonatal factors were robustly associated with lower HUI3 or SF-6D utility scores at ages 19, 28, and 35 years compared to their peers in the same cohort who did not experience these specific complications.

## Methods

### Data source and study population

**Fig. 1:**
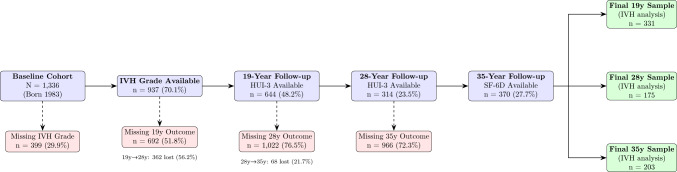
Flow Diagram: Sample Selection and Attrition in the POPS Study. Boxes show sample sizes at each stage; dashed arrows indicate exclusions due to missing data. Final analytic samples for IVH (Grade 3–4) analyses include participants with complete outcome, exposure, and covariate data. HUI-3 = Health Utilities Index Mark 3; SF-6D = Short Form 6 Dimensions; IVH = Intraventricular Hemorrhage

The POPS cohort included 94% (n = 1338) of all live-born infants in the Netherlands in 1983 with a gestational age of less than 32 weeks (VP) and/or a birth weight below 1500 g (VLBW). Table 24 shows the analytic sample by exposure and time point; Fig. 1 shows the flowchart of participants from birth through follow-up. Although 1338 infants were originally enrolled and this number is reported in earlier publications [63], the analytic dataset comprised of 1336 unique participants. During data transfer for the RECAP study, the POPS investigators discovered that one twin pair had been entered twice. The number of participants providing HRQoL data varied at each time point due to attrition [63–66].

The inclusion criteria of the POPS study allow the comparison of three distinct groups: (1) infants who were born VP & VLBW (i.e. combined effect), (2) infants who were born VP-only, and (3) infants who were VLBW-only. Gestational age was determined using data from the last menstrual period, pregnancy tests, and/or ultrasound findings.

### Outcome variables

The primary outcomes were preference-based HRQoL utility scores measured at three adult time points. The Health Utilities Index Mark 3 was administered at 19 and 28 years. The HUI3 assesses health status across eight attributes: vision, hearing, speech, ambulation, dexterity, emotion, cognition, and pain/discomfort [50]. Responses were converted into multi-attribute utility (MAU) scores using Canadian preference weights [49]. HUI3 MAU scores range from − 0.36 (worst health state) to 1.0 (full health), with 0.0 representing death [67]. At the 35-year followup, the SF-12 questionnaire was administered [52]. Responses were used to derive SF-6D utility scores using the UK preference-based algorithm developed by Brazier et al. [51]. The SF-6D describes health across six dimensions: physical functioning, role limitations, social functioning, pain, mental health, and vitality. SF-6D scores theoretically range from 0 (death) to 1.0 (perfect health), although the practical range based on the algorithm is typically narrower (e.g., ≈ 0.3 to 1.0). Secondary outcomes included binary indicators for achieving the optimal level of functioning (level 1) on each individual attribute of the HUI3 and SF-6D measures.

### Main exposures

The main independent variables represented key neonatal characteristics documented at birth or during the neonatal period: BPD: binary indicator (Yes/No) diagnosed during neonatal admission and recorded in POPS files according to contemporaneous clinical criteria aligned with Bell staging; however, POPS did not consistently record medical versus surgical management, precluding severity stratification.

We created a three-category IVH classification: No IVH (n = 1003; 75.1% of cohort), Mild IVH (Grades 1–2; n = 75; 5.6%)—subependymal hemorrhage or hemorrhage with normal ventricular size and Severe IVH (Grades 3–4; n = 129; 9.7%)—hemorrhage with ventricular dilation or parenchymal involvement is the primary exposure. A separate binary indicator comparing infants with grade 3 or 4 IVH (Yes = 1) versus those with no IVH or only grade 1 or 2 IVH (No = 0). This distinction was examined specifically because severe IVH is strongly associated with adverse long-term neurodevelopmental outcomes [31, 32], which are anticipated to have a more pronounced impact on adult HRQoL compared to milder grades. We additionally report results for IVH of any grade to facilitate comparability with prior cohort studies that did not distinguish IVH severity, while emphasizing that severity-stratified analyses are clinically more informative [68–70].

NEC: binary indicator (Yes/No), diagnosed during neonatal admission and recorded in POPS files according to contemporaneous clinical criteria aligned with Bell staging; however, POPS did not consistently record medical versus surgical management, precluding severity stratification.

Multiple Birth (MB): binary indicator (Multiple/Singleton). Any Risk Factor: a composite binary indicator (Yes/No) for having experienced at least one of BPD, IVH, NEC, or being part of a multiple birth. In all descriptive and regression analyses, the reference group comprised POPS participants who did not have the specific exposure under examination (i.e., comparisons were *within* VP/VLBW survivors rather than versus term-born controls).

### Covariates

Based on established literature between socio-demographic factors and HRQoL in preterm populations [71, 72], the following covariates were included in adjusted analyses: sex (male/female), GA (in completed weeks), birth weight (in grams), age at assessment, maternal age at birth (in years), maternal education level at birth categorized according to the International Standard Classification of Education (ISCED) into low (ISCED 0–2, reference), medium (ISCED 3–5), and high (ISCED 6–8) [73], maternal ethnicity (Caucasian/non-Caucasian, based on maternal self-report or records) and maternal marital status (married or cohabiting vs. other). Adult socioeconomic outcomes were not included as covariates because they are on the causal pathway from neonatal complications to adult HRQoL. This might bias the overall association of neonatal complications with HRQoL.

### Statistical analysis

Our primary analytical strategy involved fitting a multivariable linear regression model that simultaneously included all four neonatal complications (BPD, severe IVH, NEC, and multiple birth) to estimate their independent associations with HRQoL utility scores at each time point. This was complemented by models assessing each complication individually. To address potential biases, this core analysis was supplemented with IPW for attrition and a formal sensitivity analysis for unobserved confounding. Descriptive statistics (means, standard deviations, counts, percentages) were calculated for baseline characteristics and HRQoL outcomes. Unadjusted comparisons between exposure groups (e.g., BPD vs. no-BPD) used independent samples *t*-tests for continuous variables (utility scores, GA, BW) and *χ*^2^/chi-squared tests or Fisher’s exact test for categorical variables (e.g. sex, optimal level of functioning indicator, maternal education), as indicated by the *p*-values presented in the descriptive tables.

Our identification strategy relies on the selection-on-observables assumption to estimate robust associations rather than causal effects. That is, after controlling for a rich set of observed perinatal and maternal characteristics our findings are best viewed as robust associations rather than causal effects. The plausibility of this assumption is supported by clinical evidence suggesting that the occurrence of BPD, IVH, or NEC, while associated with risk factors like low gestational age, remains largely unpredictable for an individual infant. Specifically, numerous studies have shown that despite known risk factors, the occurrence of BPD [33, 34], IVH [35–37], or NEC [38–40] in a given infant are essentially unpredictable. This is particularly relevant since our cohort was recruited in 1983. However, because the potential for unobserved confounding can never be fully eliminated in an observational study, our primary estimates should be interpreted as robustly adjusted associations. To formally assess the potential impact of unobserved confounders, we conduct a sensitivity analysis using the method proposed by Oster [74].

Multivariable linear regression models Ordinary Least Squares (OLS) were used to estimate the adjusted association between each main exposure (BPD, IVH, NEC, MB—entered separately in different models) and the continuous HRQoL utility scores (HUI3 MAU at 19 y/28 y, SF-6D score at 35 y). Thus, our primary coefficients were derived from separate multivariable models for each exposure. We also conducted a comprehensive multivariable analysis including all four neonatal complications simultaneously to assess their independent associations.

These models included the covariates listed above. The resulting beta coefficients (*β*) represent the adjusted mean difference in utility score associated with having the exposure compared to not having it. Linear probability model was also used to model the association between exposures and the likelihood of reporting optimal functioning (level 1) on individual HUI3 and SF-6D attributes which have been show to outperform logistic regression particularly in rare events data [75–78].

Given the longitudinal nature and attrition, inverse probability weighting (IPW) have been applied in line with best practices for the POPS cohort to account for potential bias due to selective attrition. We restricted to participants with complete data on outcome and covariates and then used IPW to mitigate bias from lost follow-up. Of the original 1336 participants, follow-up data multi-attribute utility scores (MAU scores) were available for 644 (48.2%) at 19 years, 314 (23.5%) at 28 years, and 370 (27.7%) at 35 years. The 35-year follow-up involved renewed tracing and recruitment; therefore, some individuals who did not participate at 28 years returned at 35 years. We observed evidence of selective attrition across all follow-up waves (Table 1). Participants lost to follow-up were consistently more likely to have had a lower gestational age, a lower birth weight, and a mother who was younger, of non-caucasian ethnicity, and had a lower level of education (all *p* < 0.05). Neonatal complications such as BPD and NEC were not significantly associated with attrition, though individuals from a multiple birth were significantly more likely to be lost to follow-up at the 35-year wave (*p* = 0.03). Variables included in the IPW model were: sex, GA, birth weight, maternal age at birth, maternal education, maternal ethnicity, maternal marital status (Table 25).

**Table 1:** Baseline characteristics of the cohort by attrition status at each follow-up wave

Baseline characteristic	At 19 years			At 28 years			At 35 years		
	Followed-up	Attrited	*p*-value	Followed-up	Attrited	*p*-value	Followed-up	Attrited	*p*-value
	(N = 644)	(N = 692)		(N = 314)	(N = 1022)		(N = 370)	(N = 966)	
Neonatal complications, n (%)									
BPD	35 (5.4)	54 (8.0)	0.06	17 (5.4)	72 (7.2)	0.27	19 (5.1)	70 (7.4)	0.14
NEC	39 (6.1)	34 (5.0)	0.42	21 (6.7)	52 (5.2)	0.31	23 (6.2)	50 (5.3)	0.50
Severe IVH (Grades 3/4)	27 (5.6)	102 (22.4)	**< 0.001**	17 (7.3)	112 (15.9)	**< 0.001**	15 (5.6)	114 (17.0)	**< 0.001**
Multiple birth	138 (21.4)	172 (24.9)	0.14	64 (20.4)	246 (24.1)	0.18	71 (19.2)	239 (24.7)	**0.03**
Perinatal characteristics, mean (SD)									
Gestational age (weeks)	31.01 (2.46)	29.67 (3.05)	**< 0.001**	30.98 (2.34)	30.12 (2.98)	**< 0.001**	31.05 (2.37)	30.04 (2.98)	**< 0.001**
Birth weight (g)	1312.17 (293.81)	1190.14 (330.49)	**< 0.001**	1310.10 (304.49)	1230.18 (321.29)	**< 0.001**	1324.83 (297.19)	1219.90 (322.57)	**< 0.001**
Maternal characteristics									
Age at birth, mean (SD)	27.80 (4.67)	26.80 (5.01)	**< 0.001**	28.17 (4.34)	27.01 (4.99)	**< 0.001**	28.21 (4.11)	26.93 (5.09)	**< 0.001**
Non-Caucasian, n (%)	69 (10.8)	120 (17.4)	**< 0.001**	18 (5.8)	171 (16.8)	**< 0.001**	19 (5.2)	170 (17.7)	**< 0.001**
Low education	179 (38.7)	73 (60.8)	**< 0.001**	92 (37.6)	160 (47.5)	**0.03**	94 (32.3)	158 (54.3)	**< 0.001**

BPD, Bronchopulmonary dysplasia; IVH, Intraventricular hemorrhage; NEC, Necrotizing enterocolitis; Low education refers to ISCED levels 0–2. *p*-values for education level are from chi-squared tests across all three categories

#### Sensitivity analyses for attrition bias

To formally assess the robustness of our findings to potential attrition bias, we conducted three complementary sensitivity analyses: (1) Comparison of IPW-weighted versus unweighted estimates. We re-estimated all primary models with and without inverse probability weights and compared the resulting coefficients. The consistency between weighted and unweighted estimates provides evidence regarding the degree to which attrition bias affects our conclusions. (2) Best-case/worst-case imputation analysis: we bounded the potential impact of missing data by imputing missing HRQoL outcomes under two extreme scenarios: Best Case: Missing values for exposed participants (severe IVH) were imputed at the 90th percentile of observed outcomes; missing values for unexposed participants were imputed at the 10th percentile. Worst Case: Missing values for exposed participants were imputed at the 10th percentile; missing values for unexposed participants were imputed at the 90th percentile. This analysis establishes plausible bounds within which the true association likely falls. (3) Characterization of Missing Values: We created detailed tables comparing baseline characteristics between participants with complete versus missing outcome data at each time point (see Supplementary Tables S1–S5). For participants with missing IVH grade data, we coded severe IVH as missing (not ‘no severe IVH’) and excluded them from analyses/or handled via multiple imputation/or performed a sensitivity analysis coding them as not severe (Table 2).

**Table 2:** Basline characteristics POPS cohort

	Total	Missings/N (Pct)
*N* (%)	1336 (100.0)	0/1336 (0.0)
Male, *N* (%)	626 (47.0)	0/1336 (0.0)
Gestational age weeks, Mean (SD)	30.32 (2.86)	0/1336 (0.0)
Birth weight (g), Mean (SD)	1248.96 (319.11)	0/1336 (0.0)
BPD, *N* (%)	89 (6.8)	21/1336 (1.6)
IVH, *N* (%)	333 (24.9)	0/1336 (0.0)
IHV grades 3 or 4, *N* (%)	129 (13.8)	399/1336 (29.9)
NEC, *N* (%)	73 (5.5)	18/1336 (1.3)
Multiple birth, *N* (%)	310 (23.2)	0/1336 (0.0)
Maternal age, mean (SD)	27.28 (4.87)	31/1336 (2.3)
Maternal marital status, *N* (%)		
Married/co-habitating	1167 (87.7)	
Not married	163 (12.3)	6/1336 (0.4)
Maternal educational level, *N* (%)		
Low Level (ISCED 0–2)	98 (16.8)	
Medium Level (ISCED 3–5)	252 (43.3)	
High Level (ISCED 6–8)	232 (39.9)	754/1336 (56.4)
Maternal ethnicity (Caucasian), *N* (%)	1137 (85.7)	10/1336 (0.7)

BPD, Bronchopulmonary dysplasia; IVH, Intraventricular hemorrhage; NEC, Necrotizing Enterocolitis; ISCED, International Standard Classification of Education

#### Post-hoc power analysis

Given the small sample sizes in some complication groups, we calculated: The minimum detectable effect size (MDES) at 80% power and *α* = 0.05 for each exposure and time point; The achieved (post-hoc) power for the observed effect sizes and Cohen’s d standardized effect sizes to facilitate comparison across outcomes.

We initially considered a detailed analysis of the simultaneous associations and potential interactions between the four neonatal complications. However, an examination of their co– occurrence revealed this approach to be statistically infeasible due to data sparseness. While a substantial portion of the cohort experienced a single complication (28.8%), very few individuals presented with multiple conditions: only 90 participants (6.7%) had two complications, and just 12 (0.9%) had three. These small cell counts for specific combinations of morbidities would result in unstable model estimates and insufficient statistical power to reliably detect interaction effects. Consequently, our primary analysis focused on a multivariable regression model that included all four complications as independent predictors to estimate their adjusted, independent associations with long-term HRQoL.

To evaluate the long-term impact of severe IVH on HRQoL and to assess whether this impact changes over time, we employed a linear mixed-effects model. This longitudinal analysis utilized pooled data from the 19- and 28-year follow-up waves for participants with available HUI3 MAU scores. The model specified the HUI3 utility score as the dependent variable. The primary independent variables were an indicator for severe IVH (grades 3/4), a categorical variable for age at assessment (19 vs. 28 years), and an interaction term between these two variables to test for a differential association of IVH over time. The model was adjusted for sex, gestational age, birth weight, maternal age, maternal education, and maternal ethnicity as fixed effects. To account for the non-independence of repeated observations from the same individual, a random intercept was included for each participant.

### Tests for selection on unobservables: bounds under partial identification approach

Nonetheless, our identification strategy cannot completely control for all unobserved heterogeneity, so we conducted additional robustness and sensitivity analyses. We address this concern by implementing multiple additional analyses and a sensitivity test to assess the extent to which omitted unobservables might explain the observed relationships, while controlling for relevant covariates proposed by [79] and formalized by [74]. However, we were able to provide bounds for estimated association using the partial identification approach [74]. Specifically, we implemented a formal test advocated by [79] and formalized by Oster [74] to assess the degree to which omitted unobservable factors might possibly explain away the observed relation between *β*_*IVH*3*/*4_ and MAU scores following adjustment for *X* covariates.

The idea of the test is based on the assumption that the bias from observed variables contains useful data regarding the bias from unobserved variables. The coefficient of proportionality (*δ*) is understood as how substantial the impact of unobserved variables needs to be relative to the impact of observed variables for the *β*_*IVH*3*/*4_ = 0. Thus, a *δ* = 2 implies that the unobserved variables would need to be two times as substantial as the variables used in analysis to cancel the identified association (i.e. for *β*_*IVH*3*/*4_ = 0). A value of *δ* > 1 suggests that the adjusted association is likely robust [79]. To apply this test, it is required to run a linear probability regression model and to set up a maximum attainable value of *R*^2^ called *R*_*max*_ that measures the maximum variance explained by both observed and unobserved variables [74]. However, the empirical evidence suggests that *R*_*max*_ = 1 is too conservative, and Oster proposed to set *R*_*max*_ = 1.3*R*^2^ [74, where *R*^2^ measures the variability explained by observed covariates. A negative delta indicates that adding control variables increased the magnitude of the treatment effect (more negative coefficient) rather than attenuating it.

### Economic sensitivity analysis

To project the lifetime health economic burden of severe IVH, we calculated quality-adjusted life year (QALY) losses under different discount rate assumptions. We tested four discount rates: 0% (undiscounted), 1.5, 3.5 and 5%. Assuming the observed QALY decrement at age 35 persists for 45 remaining life years (to age 80), we projected lifetime QALY losses and their monetary valuations using willingness-to-pay thresholds of £20,000 and £30,000 per QALY.

All analyses were performed using Stata version 18 (Stata Corp, College Station, TX). A two-sided *p*-value of < 0.05 was considered statistically significant.

## Results

### Cohort characteristics and follow-up

The initial study cohort included data on 1336 preterm infants. Baseline characteristics for the overall cohort are presented in Table 3, while characteristics for selected subgroups assessed at follow-up are detailed in Tables 7–12. The mean GA was 30.3 weeks, and BW was 1249 g. BPD was recorded in 7%, IVH (any grade) in 25%, and NEC in 6% of the initial cohort. MB accounted for 23%. Follow-up data for HRQoL was available for subsets of the original cohort at three time points: 644 participants (48% of initial N = 1336) provided HUI3 scores at 19 years, 314 participants (23.5%) provided HUI3 scores at 28 years, and 370 participants (27.7%) provided SF-6D utility scores (derived from SF-12) at 35 years. The increase in sample size at 35 years show renewed tracing increased sample size without materially altering the composition of the analytic cohort at age 35 (Fig. 1).

**Table 3:** Adjusted association of neonatal complications with long-term health-related quality of life

Outcome measure	Adjusted coefficient	[95% CI]	*p*-value
HRQoL at 19 years (HUI3), N = 331			
BPD	0.049	[− 0.030, 0.127]	0.222
Severe IVH (grades 3–4)	− 0.096	[− 0.180, − 0.012]	0.025*
NEC	0.008	[− 0.078, 0.094]	0.857
Multiple birth	− 0.004	[− 0.050, 0.042]	0.865
HRQoL at 28 years (HUI3), N = 175			
BPD	− 0.022	[− 0.118, 0.075]	0.662
Severe IVH (grades 3–4)	− 0.126	[− 0.222, − 0.029]	0.011*
NEC	0.017	[− 0.087, 0.121]	0.747
Multiple birth	− 0.019	[− 0.084, 0.045]	0.550
HRQoL at 35 years (SF-6D), N = 203			
BPD	− 0.027	[− 0.096, 0.041]	0.434
Severe IVH (grades 3–4)	− 0.068	[− 0.132, − 0.003]	0.041*
NEC	− 0.050	[− 0.119, 0.018]	0.148
Multiple birth	− 0.025	[− 0.065, 0.015]	0.216

All models are adjusted for sex, maternal age, maternal education level, and maternal ethnicity. BPD, Bronchopulmonary dysplasia; IVH, Intraventricular hemorrhage; NEC, Necrotizing enterocolitis. The coefficient represents the adjusted mean difference in the MAU score associated with the complication compared to the reference group without the complication. [*] Statistically significant at *p* < 0.05

### Baseline characteristics by neonatal complication status at follow-up

Comparisons of baseline characteristics between participants with and without specific neonatal risk factors (BPD, IVH, NEC, MB) within the samples assessed at 19, 28, and 35 years are detailed in Tables 7–12. BPD: individuals with a history of BPD consistently had significantly lower mean GA compared to those without BPD across all follow-up assessments (*p* < 0.001). Their mean BW was also significantly lower at 19 years (*p* < 0.001), but not at 28 or 35 years. IVH: a history of IVH was associated with significantly lower mean GA (*p* < 0.001 at all ages) and lower mean BW (*p* < 0.001 at 19 y, *p* = 0.01 at 28 y, *p* = 0.03 at 35 y) compared to those without IVH. At 35 years, the IVH group had a significantly higher proportion of individuals with non-white maternal ethnicity (10.3% vs 4.0%, *p* = 0.04). Table 9 summarizes key baseline characteristics among individuals with severe IVH (grade 3 or 4) compared to those with no IVH or only grade 1–2. The subgroup with severe IVH formed approximately 5–7% of participants at each time point (N = 27 of 482 at 19 years, N = 17 of 234 at 28 years, and N = 15 of 266 at 35 years). Individuals with severe IVH had notably lower mean gestational age (e.g., 28.44 vs. 31.17 weeks at the 19-year follow-up, *p* < 0.001) and birth weight (e.g., 1195 g vs. 1311 g at 19 years, *p* = 0.05). Groups did not significantly differ with respect to maternal age, sex distribution, or maternal ethnicity. NEC: participants with a history of NEC had significantly lower mean BW at 19 years (*p* = 0.01) and 28 years (*p* = 0.03) compared to those without NEC, but mean GA did not differ significantly at any time point (Table 10). MB: individuals born as part of a multiple birth had significantly lower mean GA at 19 years (*p* = 0.01) compared to singletons, but BW and GA did not significantly differ at later assessments (Table 11). Any Risk Factor: participants with at least one of the defined risk factors (BPD, IVH, NEC, or MB) had significantly lower mean GA compared to those with none of these factors at all follow-up time points (*p* < 0.001) (Table 12).

### Unadjusted health-related quality of life outcomes

Overall mean HRQoL utility scores for the assessed cohort were 0.87 (SD 0.18) for HUI3 at 19 years, 0.89 (SD 0.17) for HUI3 at 28 years, and 0.81 (SD 0.12) for SF-6D at 35 years. Unadjusted comparisons of HRQoL utility scores and the proportion achieving optimal functioning (level 1) on individual HUI3 and SF-6D attributes are presented by risk factor status (see Tables 13–18).

*BPD* There were no statistically significant differences in mean or median HUI3 or SF-6D utility scores between participants with and without BPD at 19, 28, or 35 years. However, at 35 years, the unadjusted data (Table 13) shows BPD is associated with significantly worse mental health (*p* = 0.02); the detailed adjusted analysis (Table 19) shows a non-significant trend toward worse mental health (*p* = 0.102) (Table 4).

**Table 4:** Summary of statistically significant optimal functioning HRQoL differences by risk factors

Risk factor	Age (years)	Outcome	*p*-value
BPD	35	SF-6D physical OF	0.03
	35	SF-6D mental health OF	0.02
IVH	19	HUI3 speech OF	0.05
	19	HUI3 dexterity OF	0.01
	28	HUI3 hearing OF	0.04
IVH grade 3/4	19	HUI3 pain OF	< 0.001
	19	HUI3 ambulation OF	0.01
	19	HUI3 dexterity OF	< 0.001
	19	HUI3 MAU (mean)	0.04
	19	HUI3 MAU (median)	< 0.001
	28	HUI3 MAU (mean)	0.02
	28	HUI3 MAU (median)	0.05
	35	SF-6D physical OF	< 0.001
	35	SF-6D MAU (mean)	0.03
	35	SF-6D MAU (median)	0.02
NEC	28	HUI3 emotion OF	< 0.001
	35	SF-6D vitality OF	0.01
	35	SF-6D MAU (mean)	0.05
Multiple birth	19	HUI3 ambulation OF	0.01
	19	HUI3 cognition OF	0.03
	19	HUI3 speech OF	0.05
Any risk factor	19	HUI3 ambulation OF	0.01
	19	HUI3 dexterity OF	< 0.001
	19	HUI3 cognition OF	0.03
	35	SF-6D MAU (mean)	0.04

MAU stands for multi-attribute utility. OF, Optimal functioning

*IVH* No significant differences were observed in mean or median HUI3 or SF-6D utility scores between the IVH and no-IVH groups at any time point (*p* > 0.17 for all median/mean comparisons). At 19 years, the IVH group reported significantly less optimal functioning on HUI3 dexterity (91.7% vs 96.8%, *p* = 0.01) and speech (77.1% vs 84.9%, *p* = 0.05) (Table 14). Unadjusted mean HUI3 scores at 19 years were significantly lower among participants with grade 3 or 4 IVH than those with none or milder IVH (0.80 vs. 0.87, *p* = 0.04). Similar findings were observed at 28 years (0.80 vs. 0.89, *p* = 0.02). By 35 years, individuals with grade 3/4 IVH had a lower mean SF-6D utility (0.75 vs. 0.81, *p* = 0.03). Domain-specific results (Table 15) also indicated more pronounced difficulties with ambulation, dexterity, and pain at 19 years among those with severe IVH (Table 5).

**Table 5:** Statistically significant adjusted associations with HRQoL outcomes

Risk factor	Age (y)	Outcome	Estimate (95% CI)	*p*-value
IVH (Grade 3/4)				
IVH$_{3/4}$	19	HUI3 MAU	− 0.08 [− 0.16, − 0.00]	0.05
IVH$_{3/4}$	19	HUI3 pain OF	− 0.37 [− 0.56, − 0.18]	< 0.001
IVH$_{3/4}$	19	HUI3 ambulation OF	0.05 [0.01, 0.08]	0.01
IVH$_{3/4}$	19	HUI3 dexterity OF	− 0.12 [− 0.21, − 0.03]	0.01
IVH$_{3/4}$	28	HUI3 MAU	− 0.15 [0.28, − 0.02]	0.03
IVH$_{3/4}$	35	SF-6D MAU	− 0.07 [− 0.14, − 0.01]	0.03
IVH$_{3/4}$	35	SF-6D physical OF	0.19 [0.20, 0.77]	< 0.001
NEC				
NEC	28	HUI3 speech OF	0.084 [0.03, 0.136]	< 0.001
Multiple birth (MB)				
MB	19	HUI3 cognition OF	− 0.04 [− 0.09, − 0.001]	0.05

MAU stands for multi-attribute utility. OF, Optimal Functioning. Results at 28 and 35 are adjusted for selective attrition inverse probability weighting. Variables included in the weighting model were: sex, GA, birth weight, maternal age at birth, maternal education, maternal ethnicity and maternal marital status

*NEC* Mean and median HUI3/SF-6D scores did not differ significantly between NEC and no-NEC groups at 19 or 28 years (*p* > 0.80). At 35 years, the mean SF-6D score was lower in the NEC group, approaching statistical significance (0.76 vs 0.81, *p* = 0.05). At 35 years, they reported significantly more optimal functioning on SF-6D vitality (21.7% vs 7.2%, *p* = 0.01). Multiple Birth: No significant differences in mean or median HUI3/SF-6D utility scores were found between multiple birth individuals and singletons at any age. At 19 years, the MB group reported significantly more optimal functioning on HUI3 ambulation (2.2% vs 0.2%, *p* = 0.01) and cognition (8.7% vs 4.2%, *p* = 0.03). Any Risk Factor: Participants with at least one risk factor (BPD, IVH, NEC, or MB) did not significantly differ in HUI3 scores at 19 or 28 years compared to those with none of these factors. However, at 35 years, those with any risk factor had a significantly lower mean SF-6D utility score (0.79 vs 0.82, *p* = 0.04). At 19 years, this group reported significantly less optimal functioning on HUI3 dexterity (93.0% vs 97.9%, *p* < 0.001), but significantly more optimal functioning on HUI3 ambulation (1.6% vs 0.0%, *p* = 0.01) and cognition (7.4% vs 3.6%, *p* = 0.03).

### IPW adjusted regression analyses of HRQoL outcomes

IPW results from linear regression analyses, adjusting for potential confounders examining the association between each risk factor and HRQoL outcomes are presented in Tables 19–22. Overal, the adjusted regression analyses revealed a nuanced picture. While most neonatal complications were not independently associated with overall utility scores in adulthood, a notable exception was found for survivors with a history of severe grade 3/4 IVH, who experienced significant and persistent HRQoL decrements (Table 3).

*BPD* After adjustment, BPD was not significantly associated with overall HUI3 MAU scores at 19 or 28 years, nor with the SF-6D utility score at 35 years (*p* > 0.10 for all).

**Fig. 2:**
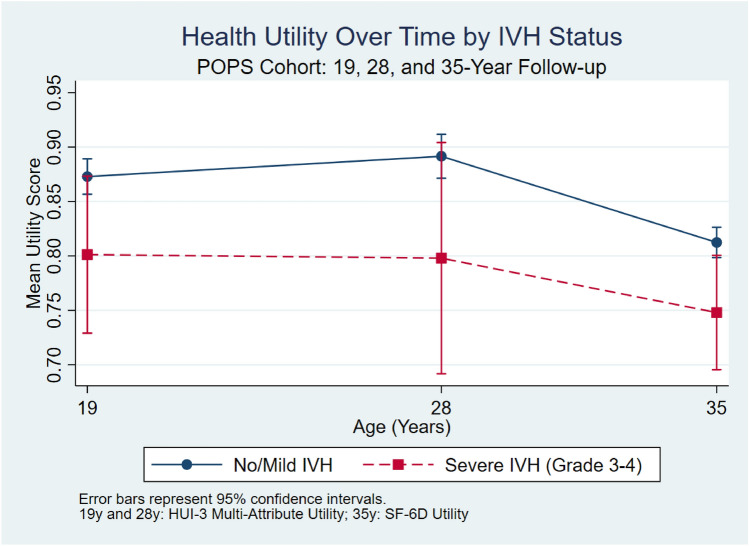
Utility over time

*IVH (any grade)* IVH was not significantly associated with overall HUI3 or SF-6D utility scores at any time point after adjustment (*p* > 0.30 for all). There were also no significant adjusted associations between IVH and optimal functioning on any individual HUI3 or SF-6D attribute (*p* ≥ 0.09 for all), including those where unadjusted differences were observed. In adjusted regression models, severe IVH was associated with statistically significant deficits in overall utility. Specifically, in Table 23 we observed the following adjusted coefficients for grade 3/4 IVH compared to no or grade 1–2 IVH: At 19 years (HUI3): *β* =  − 0.08, 95% CI [− 0.16, − 0.001], *p* = 0.05. At 28 years (HUI3): *β* =  − 0.15, 95% CI [− 0.28*,* − 0.02], *p* = 0.03. At 35 years (SF-6D): *β* =  − 0.07, 95% CI [− 0.14*,* − 0.01], *p* = 0.03. These estimates indicate that, while IVH (any grade) did not consistently predict substantially lower HRQoL overall, the subgroup with severe IVH (grades 3 or 4) experienced modest yet significant reductions in both HUI3 and SF-6D scores in adulthood (Fig. 2).

*NEC* NEC was not significantly associated with overall HUI3 or SF-6D utility scores at any time point after adjustment (*p* > 0.15 for all).

*Multiple birth* Being born as part of a MB was not significantly associated with overall HUI3 or SF-6D utility scores at any time point after adjustment (*p* > 0.15 for all). At 19 years, MB was associated with a significantly lower likelihood of optimal HUI3 cognition functioning (*β* =  − 0.04) compared to singletons. The trend towards lower likelihood of optimal ambulation at 19 years persisted but was not statistically significant (*p* = 0.06) Table 23.

In models assessing each complication individually, severe IVH was associated with a significant utility decrement at 28 years (*β* =  − 0.15, 95% CI [− 0.28, − 0.02], *p* = 0.03). This association remained significant when all neonatal complications were included in a single model (*β* =  − 0.126, 95% CI [− 0.222, − 0.029], *p* = 0.011).

### Sensitivity analysis: IPW-weighted versus unweighted comparison

To formally assess whether attrition bias affected our primary estimates, we compared unweighted and IPW-weighted regression coefficients for all exposures at each time point. Table 26 presents these results. The IPW-adjusted coefficients were similar to the unweighted estimates, with maximum |∆*β*|= 0.022. For severe IVH, all associations remained statistically significant after IPW adjustment, and the coefficients were slightly larger in magnitude at 19 and 28 years (*β* =  − 0.088 vs − 0.080 at 19y; *β* =  − 0.146 vs − 0.128 at 28y), suggesting our unweighted estimates may be conservative. This consistency strongly supports our conclusion that attrition bias had minimal impact on the primary findings.

### Best-case/worst-case sensitivity analysis

Table 27 presents the results of the best-case/worst-case sensitivity analysis for severe IVH. Our observed estimates (unweighted: *β* =  − 0.073 to − 0.128) fall between these extreme bounds and are closer to the worst-case values. If anything, selective attrition of sicker individuals may lead to *underestimation* of the true negative effects of severe IVH.

### Post-hoc power analysis

Table 28 presents the post-hoc power analysis results, including minimum detectable effect sizes (MDES) at 80% power. Severe IVH: The observed effect sizes (− 0.064 to − 0.094) approached our MDES thresholds, achieving 36–59% power. Despite borderline power, all associations reached statistical significance (*p* = 0.007–0.048), suggesting clinically meaningful effects. BPD and NEC: Observed effects were substantially smaller than MDES values, resulting in low power (5–27%). We therefore cannot rule out small effects for these complications; null findings should be interpreted as no evidence of association.

### Longitudinal mixed effects models regression analyses of HRQoL outcomes

In multivariable models adjusting for perinatal and sociodemographic factors, severe IVH (grades 3–4) was the only neonatal complication consistently and independently associated with lower MAU scores in adulthood. This association corresponded to a statistically significant utility decrement at 19 years (*β* =  − 0.096; 95% CI [− 0.180, − 0.012]), 28 years (*β* =  − 0.126; 95% CI [− 0.222, − 0.029]), and 35 years (*β* =  − 0.068; 95% CI [− 0.132, − 0.003]). In contrast, after accounting for other complications, BPD, NEC, and multiple birth status were not significantly associated with HRQoL scores at any follow-up wave.

The mixed-effects model, using 506 observations from 343 individuals, revealed a statistically significant and persistent negative main adjusted association of severe IVH on HUI3 utility scores. After adjusting for covariates, participants with a history of severe IVH had an average HUI3 score that was 0.122 points lower than those without severe IVH at the baseline age of 19 years (95% CI [-0.202, -0.042]; *p* = 0.003). The interaction term between severe IVH and age at assessment was small and not statistically significant (*β* =  − 0.009, *p* = 0.849), indicating that this utility decrement did not significantly change between ages 19 and 28.

### Bounds

To assess the robustness of our estimates to potential omitted variable bias, we implemented the method developed by Oster (2019). We calculated the index of proportional selection, *δ*, which represents the degree of selection on unobserved variables relative to observed covariates that would be required to reduce the estimated association of IVH to zero.

For the analysis at age 19, the calculated *δ* was − 0.99. A negative value for *δ* indicates that our observed control variables (e.g., gestational age, birth weight) were acting as suppressors. In other words, accounting for them *increased* the magnitude of the negative association between severe IVH and HRQoL. For an unobserved variable to explain away our finding, it would have to be correlated with IVH and HRQoL in the opposite direction to our main controls, a scenario considered less likely and which therefore strengthens our confidence in the result. In the model for health utility at age 28, we found that the result was moderately robust to potential unobserved confounding. The analysis yielded a *δ* of 0.41. This value implies that unobserved factors would need to be 41% as strongly correlated with both severe IVH and HUI as the included covariates to nullify the estimated association.

For the health utility outcome at age 35 (SF-6D), the robustness of the estimate increased. The calculated *δ* was 0.71, indicating that the influence of unobserved confounders would need to be 71% as strong as that of the observed controls to explain away the association. This suggests that even though the primary estimate was not statistically significant at conventional levels, it is reasonably robust to omitted variable bias. Oster’s coefficient-stability diagnostics indicate that plausible omitted-variable structures are unlikely to nullify these estimates—particularly the adolescent result, where unobservables would have to act in the opposite direction to the observables.

### Economic sensitivity analysis: discount rate variation

Table 28 presents the projected lifetime QALY losses and monetary valuations for severe IVH under different discount rate assumptions.

## Discussion

Our analysis reveals that among the major neonatal complications studied, severe IVH is the most significant independent predictor of diminished long-term HRQoL into the fourth decade of life. In our fully adjusted model accounting for BPD, NEC, and multiple birth status, severe IVH was associated with a utility decrement of 0.126 (*p* = 0.011) at age 28. This deficit is not only statistically significant but also clinically meaningful, substantially exceeding the established minimally important difference for both the HUI3 and SF-6D instruments. The lack of a significant independent association for BPD and NEC in our fully adjusted models suggests that their long-term impact on HRQoL may be less pronounced or potentially mediated by other factors once the profound consequence of a severe brain injury like IVH is accounted for. This finding has critical implications for health economic evaluations: cost-utility analyses of perinatal interventions must specifically account for the substantial and persistent qualityof-life losses attributable to severe IVH, as failing to do so may underestimate the long-term value of neuroprotective strategies.

To the best of our knowledge, this study is among the first to specifically examine the independent association of all common severe neonatal complications (BPD, IVH, NEC) and MB status with preference-based HRQoL utility scores in early and mid-adulthood, within a large, population-based cohort of VP/VLBW survivors, using data from the well-characterized Dutch POPS cohort. Our primary finding is that, after adjusting for important perinatal and socio-demographic confounders, none of these specific neonatal risk factors were consistently associated with statistically significant deficits in overall HUI3 or SF-6D utility scores compared to their peers who did not experience these specific conditions.

However, our analysis focusing specifically on severe intraventricular hemorrhage (IVH grade 3 or 4) versus no or mild IVH (grade 1–2) highlights an important exception to the general null findings for overall utility. The subgroup with severe IVH demonstrated significantly lower unadjusted HUI3 and SF-6D scores at all adult follow-up points (19, 28, and 35 years). Adjusted analyses, controlling for confounders, confirmed these modest but potentially meaningful decrements in overall utility scores. Specifically, severe IVH was associated with adjusted HUI3 score reductions of MAU scores at all assessment timepoints. From a health economics perspective, these decrements are noteworthy as they exceed the commonly cited minimally important difference threshold of 0.03 for the HUI3 instrument [80–87].

The longitudinal analysis confirms that severe neonatal IVH is associated with a substantial and enduring deficit in health-related quality of life into early adulthood. The mixed-effects model demonstrates a clinically meaningful utility decrement of approximately 0.12 points, a finding that is robust after controlling for key perinatal and sociodemographic factors. The lack of a significant interaction between severe IVH and age at assessment is a critical finding; it suggests that the gap in HRQoL established by age 19 neither narrows through potential adaptation nor worsens significantly by age 28. This persistence of the utility decrement underscores the lifelong burden conferred by severe neonatal brain injury and reinforces the need for sustained clinical and social support for these survivors. For health economic evaluations, this result justifies applying a consistent and substantial quality-of-life penalty in models assessing the long-term cost-effectiveness of interventions aimed at preventing severe IVH.

These findings align with the extensive pediatric follow-up literature indicating that higher grade IVH is a robust predictor of adverse long-term neurodevelopmental outcomes [31, 32]. Metaanalyses confirm a dose-dependent effect, with severe IVH (Grade 3–4) conferring substantially higher odds (e.g., ∼4–5 times higher) of moderate-to-severe neurodevelopmental impairment, cerebral palsy, and cognitive/motor deficits by early childhood compared to those with no or mild IVH [88, 89]. While milder grades (1–2) often do not result in significant differences in cognitive or behavioral outcomes by late adolescence [90], severe neonatal brain injury, including Grade 3–4 IVH, is clearly linked to poorer neuropsychological outcomes and functional impairment persisting into adulthood [91]. Our results extend these findings by demonstrating a corresponding impact on preference-based HRQoL utility scores. It is important to note, however, that even among survivors of severe IVH with complications like post-hemorrhagic ventricular dilatation, there is considerable variability in long-term HRQoL, with some individuals achieving nearnormal scores while others experience profound deficits, particularly in physical domains [92]. From a health economic standpoint, these results strongly caution against using a single HRQoL decrement for “any IVH” in cost-effectiveness models or policy decisions concerning VP/VLBW survivors. Distinguishing outcomes based on IVH severity is crucial for accurately estimating the lifetime burden of disease and the potential value of interventions. Strategies aimed at preventing severe IVH or mitigating its consequences could yield substantial long-term benefits, not only by improving neurodevelopmental function but also by enhancing overall adult quality of life as captured by preference-based utility measures.

Our findings are consistent with evidence from other large longitudinal cohorts showing that (i) VP/VLBW or extremely preterm birth is associated with lower HRQoL compared with term-born controls into adolescence and adulthood, and (ii) within preterm survivors, the largest and most persistent HRQoL decrements tend to concentrate among those with major neurosensory or neurodevelopmental impairment rather than being uniformly distributed across all neonatal diagnoses. For example, the Bavarian Longitudinal Study reported lower HUI3 utilities among VP/VLBW participants than term controls at ages 13 and 26, with parental reports indicating worsening over time particularly in emotion and pain domains [93]. In the EPICure program, parent-reported HUI-based utilities in childhood showed clinically meaningful limitations, and an epoch comparison reported lower HRQoL among extremely preterm children born in 2006 compared with 1995, with speech and dexterity contributing strongly to the decline—domains closely aligned with utility scoring [94–96]. Similarly, post-surfactant era cohorts have reported broadly comparable self-perceived HRQoL to controls in late adolescence on average, but with domain-specific differences (e.g., dexterity/physical functioning) that mirror the types of signals we observe for severe IVH [97]. Together, these studies support two implications for health economic evaluation: average utilities for VP/VLBW survivors may mask substantial heterogeneity, and severity markers of neurological injury are particularly important for parameterizing long-term QALY impacts.

The well-established literature shows that preterm birth itself (often VP/VLBW compared to term birth) is associated with lower HRQoL scores in adulthood [7, 61]. A related design consideration is the choice of comparison group. Our analyses compare exposed versus unexposed individuals *within the VP/VLBW survivor cohort* to estimate the incremental HRQoL burden attributable to specific neonatal complications *beyond* the baseline impact of prematurity itself. This contrast is the most directly relevant for economic evaluations that compare interventions *within* neonatal intensive care (e.g., strategies that reduce severe IVH among VP/VLBW infants). By contrast, comparisons to term-born controls would estimate a *total* burden that conflates prematurity and complication effects and answers a different policy question. For context, major cohorts comparing VP/VLBW individuals with term-born controls consistently report lower HRQoL into adulthood, indicating that the overall burden of VP/VLBW remains substantial even when incremental complication effects are small. Our results suggest that while being born VP/VLBW carries a substantial baseline risk for reduced adult HRQoL, the additional significant burden conferred by these specific complications on overall preference-based utility scores, when compared to other VP/VLBW survivors, may be less pronounced or consistently detectable than perhaps anticipated. Several factors might contribute to this observation. First, the significant underlying burden of VP/VLBW status on multiple domains might indeed”swamp” the specific additional impact of individual complications when summarized into a single utility score, especially using generic instruments [98] or due to instruments’ psychometric properties [53, 54]. Second, long-term follow-up allows for potential resilience and adaptation mechanisms to emerge [99–101]. Individuals may adapt to functional limitations, or develop coping strategies that mitigate the impact on their overall subjective well-being, a phenomenon known as the “disability paradox” [99]. Furthermore, the comparison group (survivors without the specific complication) may themselves have lower-than-average HRQoL, narrowing the observable difference. Null findings in overall utility may reflect *measurement properties* of generic preference-based instruments. Both HUI3 and SF-6D summarize multiple domains into a single index and can exhibit ceiling effects in community-dwelling samples; subtle executive, behavioral, or participation limitations that are clinically salient in VP/VLBW survivors may not shift the overall utility score if impacts are modest, compensated by strengths in other domains, or fall outside instrument emphasis. Fourth, historical measurement may introduce *exposure misclassification* (e.g., incomplete ascertainment or grading of IVH in the early ultrasound era), which would bias estimates toward the null. Finally, survivorship and selective participation can attenuate between-group differences even after IPW if the most severely affected survivors are under-represented at adult follow-up.

Despite the null findings for overall utility scores, our analyses revealed several statistically significant associations at the level of specific HRQoL domains, highlighting the importance of a multi-faceted assessment. Importantly, the domain-level results help explain why severe IVH produced consistent utility decrements whereas other morbidities did not. Severe IVH was associated with deficits in domains that carry substantial weight in preference-based scoring (e.g., pain, dexterity/physical functioning), which plausibly drives the observed reductions in overall HUI3/SF-6D utilities. In contrast, for BPD and NEC we observed scattered domain signals without a consistent pattern across instruments and time points, which may be insufficient to shift the aggregate utility index. This distinction matters for decision modeling: where interventions primarily affect a small number of functional domains, analysts should consider whether those domains are strongly reflected in the chosen utility instrument and whether domain-specific outcomes (and costs) need explicit representation alongside utilities. The association of multiple birth status with poorer cognitive function (HUI3) at 19 years is also consistent with studies reporting subtle cognitive and developmental differences in preterm multiples compared to singletons [102–104]. The lack of a significant overall association between IVH (all grades combined) and HRQoL outcomes might be explained by the grouping of mild (grades I/II) and severe (grades III/IV) hemorrhages. Severe IVH is much more strongly predictive of major neurodevelopmental impairment [31, 32], and future analyses focusing specifically on this subgroup, if sample size permits, could yield different results regarding long-term HRQoL utility.

### Robustness of findings

Our sensitivity analyses substantially strengthen confidence in the primary findings. First, the comparison of IPW-weighted versus unweighted estimates demonstrated remarkable consistency, with all coefficient changes less than 0.02 in magnitude. The fact that IPW estimates for severe IVH were slightly *larger* at 19 and 28 years suggests that, if anything, our primary (unweighted) estimates may be conservative.

Second, the best-case/worst-case analysis established that our observed estimates fall within plausible bounds and are closer to the worst-case scenario, indicating our findings are not contingent on optimistic assumptions about missing data. This pattern suggests that selective attrition of sicker individuals may actually lead to underestimation of the true effect.

Third, the post-hoc power analysis clarifies the interpretation of our findings. For severe IVH, we achieved 36–59% power and detected statistically significant associations at all time points, suggesting the true effect is clinically meaningful. For BPD and NEC, however, our study was underpowered to detect effects smaller than 0.07–0.12 utility units. We therefore cannot exclude the possibility of small but clinically relevant effects for these complications; our null findings should be interpreted as “no evidence of association” rather than definitive “no association.”

Finally, the Oster bounds analysis provides reassurance regarding unobserved confounding. The negative delta at age 19 is particularly noteworthy: it indicates that controlling for observed confounders strengthened the IVH-HRQoL association, suggesting that additional unobserved confounders with similar properties would further strengthen rather than attenuate our findings.

Our economic sensitivity analysis demonstrates that the health economic burden of severe IVH might be substantial, even with the more conservative 5% discount rate, the lifetime impact remains 1.21 QALYs. These projections provide benchmarks for evaluating preventive interventions. This economic framing underscores the potential value of investing in neuroprotective strategies and supports the prioritization of research aimed at reducing the incidence of severe IVH in preterm populations. The absence of strong, consistent overall utility decrements specifically attributable to BPD, NEC, or MB beyond the effect of VP/VLBW birth suggests caution when applying large, specific utility penalties for these conditions in cost-effectiveness models comparing interventions within the VP/VLBW population, especially if based on comparisons to term-born controls. This is particularly true when the comparator is another intervention within the VP/VLBW population. However, our domain-specific findings suggest that while the overall utility score may not be significantly lower, the costs associated with managing these specific morbidities over a lifetime are likely to be substantial. Economic models should therefore focus on capturing these specific cost drivers, even if a distinct utility penalty is not applied for conditions other than severe IVH. Using decrements derived from the general VP/VLBW population average [61] might be more appropriate as a baseline, with adjustments potentially considered based on the presence of severe functional impairment rather than solely the neonatal diagnosis. However, the significant domain-specific findings strongly reinforce the need for targeted, long-term support tailored to the known sequelae of these conditions. These domainspecific morbidities likely drive differences in healthcare utilization, educational support needs, and societal costs that are not fully captured by overall HRQoL utility scores [105, 106]. Therefore, resource allocation and intervention strategies must consider these nuanced effects.

These findings have implications for health policy and resource allocation. The substantial lifetime burden, which remains significant even when using a more conservative utility decrement (− 0.068, yielding 1.96 discounted QALYs lost) or a lower discount rate (1.5%, yielding 5.05 discounted QALYs lost) (see Table 29 for comprehensive sensitivity analysis), underscores the economic imperative to address this neonatal complication. This valuation provides justification for prioritizing research funding for novel neuroprotective strategies, supporting the implementation of evidence-based clinical practices in neonatal intensive care, and ensuring that the long-term, patient-centered consequences of severe IVH are central to future health economic models. This analysis demonstrates that investments in preventing neonatal brain injury are not merely costs, but high-value investments in decades of future health and well-being. These projections are illustrative and not intended as contemporaneous cost-effectiveness thresholds. Changes in survival, severity mix, and long-term support services in modern cohorts may alter both the prevalence of severe IVH and its average lifetime utility impact.

This study leverages the significant strengths of the POPS cohort, including its population-based design mitigating selection bias, prospective data collection from birth, long-term longitudinal follow-up into mid-adulthood, and the use of internationally recognized, validated preference-based HRQoL measures suitable for economic evaluation [49, 51, 53, 62]. Nevertheless, several limitations must be acknowledged. The methods used here are based on an assumption of selection on observables that cannot be verified with observed data [107, 108]. Our findings are best viewed as robust associations rather than causal effects. Although clinical and biological evidence indicates that BPD, IVH, and NEC behave largely as exogenous shocks—and we have adjusted for several key confounders—the possibility of residual or unmeasured confounding cannot be entirely ruled out. Thus, this study may not fully account for all potential confounding variables that could influence the relationship between VP/VLBW status and HRQoL outcomes in early adulthood. POPS study had substantial attrition which occurred over the 35-year follow-up period [64]. Our comprehensive sensitivity analyses—including IPW comparison and best-case/worst-case analyses—demonstrated that attrition bias had minimal impact on our primary findings, with all conclusions remaining unchanged after adjustment [109, 110].

Second, the sample sizes for specific complication groups, particularly BPD (N≈17–35 depending on wave) and NEC (N ≈ 21–39), were relatively small at the adult follow-up assessments, limiting statistical power to detect potentially modest but clinically meaningful differences in HRQoL utility (e.g., differences less than the commonly cited minimally important difference thresholds for HUI3/SF-6D of 0.03–0.05) [80–87, 111]. Our post-hoc power analysis (Table 28) confirms that we were adequately powered (36–59%) to detect the moderate effects observed for severe IVH, but underpowered (5–27%) for BPD and NEC. Null findings for these complications should therefore be interpreted as “no evidence of association” rather than definitive absence of effect. Third, as noted, IVH was analyzed combining all grades due to sample size constraints for severe IVH.

Fourth, the cohort was born in 1983 and therefore reflects an era of neonatal intensive care that predates several practices now considered standard (e.g., widespread antenatal corticosteroids, *status and indicator severe IVH* non-invasive ventilation strategies, caffeine therapy, tighter oxygen saturation targeting, and bundled neuroprotection/NEC-prevention pathways). These advances have changed both (i) the incidence and severity distribution of neonatal morbidities and (ii) the case-mix of survivors, because improved survival at the lowest gestational ages increases the proportion of infants at risk of chronic morbidity. As a result, the absolute prevalence of BPD, IVH, and NEC—and their downstream sequelae—may differ in contemporary cohorts, so the transportability of our point estimates requires caution.

Importantly, however, evidence from more recent comparisons suggests that improved survival has not translated into uniform improvements in later functional and quality-of-life outcomes. For example, school-age and adolescent follow-up of extremely preterm cohorts has shown persistent (and in some settings worsening) profiles in domains closely related to utility measurement (e.g., motor function; speech/dexterity; neurodevelopmental/behavioral outcomes), despite substantial changes in neonatal care [95, 96, 112–114]. These findings support the continued relevance of long-term HRQoL evidence for decision models, particularly for complications that represent structural injury (e.g., severe IVH) where the underlying pathophysiology is unlikely to be eliminated even if incidence changes. At the same time, contemporary cohorts may experience different patterns of morbidity clustering and survivorship, so future work should replicate these analyses in newer cohorts as long-term follow-up accrues.

Fifth, our dataset does not distinguish between medical and surgical NEC, nor does it include information on VP shunt placement following post-hemorrhagic hydrocephalus. These distinctions may be important for understanding the full spectrum of long-term outcomes, and we recommend that future data collection efforts include these variables.

While modern clinical practice may differ from practice during the range of birth years across the cohort considered, however, it is plausible that the underlying biological impacts of VP/VLBW remains informative as the evidence shows that despite changes in practices there is no improvements in outcome [95, 96, 112–114]. Future research is needed to assess whether improvements in care have modified long-term outcomes using more recent data. Sixth, being a single-country study in a high-income setting (the Netherlands), findings may not directly apply to different healthcare systems or socioeconomic contexts. Analyses of domain-level optimal functioning outcomes were exploratory and involved multiple hypothesis tests. We therefore interpret domain-specific *p*-values cautiously.

## Conclusion

In this population-based cohort of Dutch VP/VLBW survivors born in 1983, experiencing BPD, NEC, or being part of a MB during the neonatal period was generally not associated with significantly lower overall preference-based HRQoL utility scores in early adulthood compared to peers without these specific conditions. Severe IVH (grade 3/4) was associated with clinically significant decrements in HUI3 at 19 and 28 and in SF-6D at 35 years. These findings were robust to comprehensive sensitivity analyses including IPW adjustment for attrition, best-case/worstcase imputation scenarios, and assessment of unobserved confounding. Overall, these findings highlight the complexity of long-term outcomes after VP/VLBW birth and suggest that while overall utility may appear similar between subgroups, targeted support addressing specific functional limitations remains crucial. Health economic evaluations should consider these nuances rather than applying uniform utility decrements based solely on the presence of these neonatal complications. Further research in contemporary cohorts, potentially with larger samples for specific complications and analysis of IVH severity, is needed.

## Data Availability

The data that support the findings of this study are not available due to restrictions related to participant privacy and the informed consent under which the data were collected.

## Appendix 1

See Tables 6, 7, 8, 9, 10, 11 and 12.

**Table 6:** Sensitivity analysis of the estimated associations of severe IVH on adult health utility

Outcome	OLS		Oster (2019)	
	Coefficient (*β *)	R-squared	*δ*	Observations
HUI-3 at age 19	− 0.080	0.039	− 0.99	331
HUI-3 at age $28^{\textrm{a}}$	− 0.075	0.41	0.365	163
SF-6D at age 35	− 0.070	0.037	0.710	187

This table summarizes the results of two sensitivity analyses testing the robustness of the estimated association of severe IVH on adult health utility scores to potential unobserved confounding. All underlying models control for child’s sex, maternal age, maternal education, and maternal ethnicity. The Oster (2019) *δ * quantifies the degree of selection on unobservables relative to selection on observables that would be necessary to reduce the estimated coefficient to zero. A common, conservative threshold for a robust result is *|δ | > 1*.

$^{\textrm{a}}$ The sensitivity analyses for the age 28 outcome were conditioned on the availability of the age 19 outcome, resulting in a smaller sample size (N = 163) and a slightly different OLS coefficient and R-squared than the main regression model for that age (which had N = 175). The values shown are from the models used for the sensitivity tests

**Table 7:** Baseline characteristics by BPD status

	BPD	No-BPD	Total	*p*-value	Missings/*N* (Pct)
At 19 years					
*N* (%)	35 (5.4)	609 (94.6)	644 (100.0)		0/644 (0.0)
Child sex, *N* (%)					
Male	25 (71.4)	269 (44.2)	294 (45.7)	<0.001	0/644 (0.0)
Female	10 (28.6)	340 (55.8)	350 (54.3)		
Gestational age (weeks), mean (SD)	28.76 (1.71)	31.14 (2.44)	31.01 (2.46)	<0.001	0/644 (0.0)
Birth weight (g), mean (SD)	1174.29 (228.96)	1320.09 (295.31)	1312.17 (293.81)	<0.001	0/644 (0.0)
Maternal age at birth (years), mean (SD)	27.05 (4.29)	27.84 (4.69)	27.80 (4.67)	0.33	12/644 (1.9)
Maternal education level at birth, *N* (%)					
Low level (ISCED 0–2)	13 (43.3)	166 (38.4)	179 (38.7)		
Medium level (ISCED 3–5)	10 (33.3)	186 (43.1)	196 (42.4)	0.56	182/644 (28.3)
High level (ISCED 6–8)	7 (23.3)	80 (18.5)	87 (18.8)		
Maternal ethnicity, *N* (%)					
Caucasian	33 (94.3)	536 (88.9)	569 (89.2)		
Non-Caucasian	2 (5.7)	67 (11.1)	69 (10.8)	0.32	6/644 (0.9)
At 28 years					
*N* (%)	17 (5.4)	297 (94.6)	314 (100.0)		0/314 (0.0)
Child sex, *N* (%)					
Male	10 (58.8)	109 (36.7)	119 (37.9)	0.07	0/314 (0.0)
Female	7 (41.2)	188 (63.3)	195 (62.1)		
Gestational age (weeks), mean (SD)	28.87 (1.66)	31.10 (2.31)	30.98 (2.34)	<0.001	0/314 (0.0)
Birth weight (g), mean (SD)	1223.24 (255.47)	1315.07 (306.68)	1310.10 (304.49)	0.23	0/314 (0.0)
Maternal age at birth (years), mean (SD)	28.10 (4.40)	28.18 (4.34)	28.17 (4.34)	0.94	9/314 (2.9)
Maternal education level at birth, *N* (%)					
Low level (ISCED 0–2)	7 (41.2)	85 (37.3)	92 (37.6)		
Medium level (ISCED 3–5)	6 (35.3)	97 (42.5)	103 (42.0)	0.84	69/314 (22.0)
High level (ISCED 6–8)	4 (23.5)	46 (20.2)	50 (20.4)		
Maternal ethnicity, *N* (%)					
Caucasian	17 (100.0)	276 (93.9)	293 (94.2)		
Non-Caucasian	0 (0.0)	18 (6.1)	18 (5.8)	0.29	3/314 (1.0)
At 35 years					
*N* (%)	19 (5.1)	351 (94.9)	370 (100.0)		0/370 (0.0)
Child sex, *N* (%)					
Male	12 (63.2)	149 (42.5)	161 (43.5)	0.08	0/370 (0.0)
Female	7 (36.8)	202 (57.5)	209 (56.5)		
Gestational age (weeks), mean (SD)	28.79 (1.84)	31.17 (2.34)	31.05 (2.37)	<0.001	0/370 (0.0)
Birth weight (g), mean (SD)	1216.58 (165.87)	1330.69 (301.71)	1324.83 (297.19)	0.10	0/370 (0.0)
Maternal age at birth (years), mean (SD)	27.74 (4.58)	28.23 (4.09)	28.21 (4.11)	0.61	8/370 (2.2)
Maternal education level at birth, *N* (%)					
Low level (ISCED 0–2)	6 (35.3)	88 (32.1)	94 (32.3)		
Medium level (ISCED 3–5)	7 (41.2)	130 (47.4)	137 (47.1)	0.88	79/370 (21.4)
High level (ISCED 6–8)	4 (23.5)	56 (20.4)	60 (20.6)		
Maternal ethnicity, *N* (%)					
Caucasian	19 (100.0)	327 (94.5)	346 (94.8)		
Non-Caucasian	0 (0.0)	19 (5.5)	19 (5.2)	0.29	5/370 (1.4)

BPD, Bronchopulmonary dysplasia; ISCED, International Standard Classification of Education

**Table 8:** Baseline characteristics by IVH status

	IVH (grade 1 to 4)	No-IVH	Total	*p*-value	Missings/*N* (Pct)
At 19 years					
*N* (%)	109 (16.9)	535 (83.1)	644 (100.0)		0/644 (0.0)
Child sex, *N* (%)					
Male	48 (44.0)	246 (46.0)	294 (45.7)	0.71	0/644 (0.0)
Female	61 (56.0)	289 (54.0)	350 (54.3)		
Gestational age (weeks), mean (SD)	29.57 (2.00)	31.31 (2.45)	31.01 (2.46)	<0.001	0/644 (0.0)
Birth weight (g), mean (SD)	1236.72 (303.24)	1327.54 (289.74)	1312.17 (293.81)	<0.001	0/644 (0.0)
Maternal age at birth (years), mean (SD)	27.86 (4.50)	27.78 (4.70)	27.80 (4.67)	0.88	12/644 (1.9)
Maternal education level at birth, *N* (%)					
Low level (ISCED 0–2)	23 (30.7)	156 (40.3)	179 (38.7)		
Medium level (ISCED 3–5)	37 (49.3)	159 (41.1)	196 (42.4)	0.28	182/644 (28.3)
High level (ISCED 6–8)	15 (20.0)	72 (18.6)	87 (18.8)		
Maternal ethnicity, *N* (%)					
Caucasian	90 (84.1)	479 (90.2)	569 (89.2)		
Non-Caucasian	17 (15.9)	52 (9.8)	69 (10.8)	0.06	6/644 (0.9)
At 28 years					
*N* (%)	59 (18.8)	255 (81.2)	314 (100.0)		0/314 (0.0)
Child sex, *N* (%)					
Male	21 (35.6)	98 (38.4)	119 (37.9)	0.69	0/314 (0.0)
Female	38 (64.4)	157 (61.6)	195 (62.1)		
Gestational age (weeks), mean (SD)	29.53 (2.02)	31.31 (2.28)	30.98 (2.34)	<0.001	0/314 (0.0)
Birth weight (g), mean (SD)	1211.86 (301.62)	1332.83 (301.19)	1310.10 (304.49)	0.01	0/314 (0.0)
Maternal age at birth (years), mean (SD)	28.06 (4.41)	28.20 (4.33)	28.17 (4.34)	0.82	9/314 (2.9)
Maternal education level at birth, *N* (%)					
Low level (ISCED 0–2)	13 (28.3)	79 (39.7)	92 (37.6)		
Medium level (ISCED 3–5)	24 (52.2)	79 (39.7)	103 (42.0)	0.26	69/314 (22.0)
High level (ISCED 6–8)	9 (19.6)	41 (20.6)	50 (20.4)		
Maternal ethnicity, *N* (%)					
Caucasian	53 (91.4)	240 (94.9)	293 (94.2)		
Non-Caucasian	5 (8.6)	13 (5.1)	18 (5.8)	0.31	3/314 (1.0)
At 35 years					
*N* (%)	69 (18.6)	301 (81.4)	370 (100.0)		0/370 (0.0)
Child sex, *N* (%)					
Male	29 (42.0)	132 (43.9)	161 (43.5)	0.78	0/370 (0.0)
Female	40 (58.0)	169 (56.1)	209 (56.5)		
Gestational age (weeks), mean (SD)	29.41 (1.82)	31.42 (2.33)	31.05 (2.37)	<0.001	0/370 (0.0)
Birth weight (g), mean (SD)	1255.29 (299.51)	1340.77 (294.85)	1324.83 (297.19)	0.03	0/370 (0.0)
Maternal age at birth (years), mean (SD)	27.38 (4.46)	28.40 (4.01)	28.21 (4.11)	0.06	8/370 (2.2)
Maternal education level at birth, *N* (%)					
Low level (ISCED 0–2)	15 (26.8)	79 (33.6)	94 (32.3)		
Medium level (ISCED 3–5)	30 (53.6)	107 (45.5)	137 (47.1)	0.52	79/370 (21.4)
High level (ISCED 6–8)	11 (19.6)	49 (20.9)	60 (20.6)		
Maternal ethnicity, *N* (%)					
Caucasian	61 (89.7)	285 (96.0)	346 (94.8)		
Non-Caucasian	7 (10.3)	12 (4.0)	19 (5.2)	0.04	5/370 (1.4)

IVH, Intraventricular Hemorrhage; ISCED, International Standard Classification of Education

**Table 9:** Baseline characteristics by IVH Grade 3 or 4 status

	Grade 1 or 2 or no IVH	IVH Grade 3 or 4	Total	*p*-value	Missings/*N* (Pct)
At 19 years					
*N* (%)	455 (94.4)	27 (5.6)	482 (100.0)		162/644 (25.2)
Child sex, *N* (%)					
Male	199 (43.7)	12 (44.4)	211 (43.8)	0.94	0/644 (0.0)
Female	256 (56.3)	15 (55.6)	271 (56.2)		
Gestational age (weeks), mean (SD)	31.17 (2.42)	28.44 (1.56)	31.02 (2.46)	<0.001	0/644 (0.0)
Birth weight (g), mean (SD)	1310.93 (296.02)	1195.00 (292.51)	1304.43 (296.73)	0.05	0/644 (0.0)
Maternal age at birth (years), mean (SD)	27.88 (4.72)	27.44 (3.89)	27.85 (4.67)	0.64	12/644 (1.9)
Maternal education level at birth, *N* (%)					
Low level (ISCED 0–2)	120 (37.4)	3 (15.8)	123 (36.2)		
Medium level (ISCED 3–5)	139 (43.3)	11 (57.9)	150 (44.1)	0.16	182/644 (28.3)
High level (ISCED 6–8)	62 (19.3)	5 (26.3)	67 (19.7)		
Maternal ethnicity, *N* (%)					
Caucasian	401 (88.5)	23 (85.2)	424 (88.3)		
Non-Caucasian	52 (11.5)	4 (14.8)	56 (11.7)	0.60	6/644 (0.9)
At 28 years					
*N* (%)	217 (92.7)	17 (7.3)	234 (100.0)		80/314 (25.5)
Child sex, *N* (%)					
Male	76 (35.0)	7 (41.2)	83 (35.5)	0.61	0/314 (0.0)
Female	141 (65.0)	10 (58.8)	151 (64.5)		
Gestational age (weeks), mean (SD)	31.11 (2.23)	28.13 (1.66)	30.89 (2.32)	<0.001	0/314 (0.0)
Birth weight (g), mean (SD)	1314.86 (319.01)	1163.53 (275.19)	1303.87 (317.95)	0.06	0/314 (0.0)
Maternal age at birth (years), mean (SD)	28.22 (4.29)	28.46 (3.61)	28.24 (4.24)	0.82	9/314 (2.9)
Maternal education level at birth, *N* (%)					
Low level (ISCED 0–2)	60 (36.1)	3 (21.4)	63 (35.0)		
Medium level (ISCED 3–5)	73 (44.0)	6 (42.9)	79 (43.9)	0.31	69/314 (22.0)
High level (ISCED 6–8)	33 (19.9)	5 (35.7)	38 (21.1)		
Maternal ethnicity, *N* (%)					
Caucasian	203 (94.0)	16 (94.1)	219 (94.0)		
Non-Caucasian	13 (6.0)	1 (5.9)	14 (6.0)	0.98	3/314 (1.0)
At 35 years					
*N* (%)	251 (94.4)	15 (5.6)	266 (100.0)		104/370 (28.1)
Child sex, *N* (%)					
Male	104 (41.4)	5 (33.3)	109 (41.0)	0.54	0/370 (0.0)
Female	147 (58.6)	10 (66.7)	157 (59.0)		
Age (weeks), mean (SD)	31.17 (2.27)	28.10 (1.66)	31.00 (2.35)	<0.001	0/370 (0.0)
Birth weight (g), mean (SD)	1318.19 (304.46)	1203.00 (301.97)	1311.69 (304.92)	0.16	0/370 (0.0)
Maternal age at birth (years), mean (SD)	28.30 (4.02)	27.14 (2.88)	28.23 (3.97)	0.28	8/370 (2.2)
Maternal education level at birth, *N* (%)					
Low level (ISCED 0–2)	61 (31.6)	2 (14.3)	63 (30.4)		
Medium level (ISCED 3–5)	90 (46.6)	7 (50.0)	97 (46.9)	0.30	79/370 (21.4)
High level (ISCED 6–8)	42 (21.8)	5 (35.7)	47 (22.7)		
Maternal ethnicity, *N* (%)					
Caucasian	236 (94.4)	14 (93.3)	250 (94.3)		
Non-Caucasian	14 (5.6)	1 (6.7)	15 (5.7)	0.86	5/370 (1.4)

IVH, Intraventricular Hemorrhage; ISCED, International Standard Classification of Education

**Table 10:** Baseline characteristics by NEC status

	NEC	No-NEC	Total	*p*-value	Missings/*N* (Pct)
At 19 years					
*N* (%)	39 (6.1)	605 (93.9)	644 (100.0)		0/644 (0.0)
Child sex, *N* (%)					
Male	17 (43.6)	277 (45.8)	294 (45.7)	0.79	0/644 (0.0)
Female	22 (56.4)	328 (54.2)	350 (54.3)		
Gestational age (weeks), mean (SD)	30.75 (2.28)	31.03 (2.48)	31.01 (2.46)	0.50	0/644 (0.0)
Birth weight (g), mean (SD)	1199.10 (293.40)	1319.45 (292.58)	1312.17 (293.81)	0.01	0/644 (0.0)
Maternal age at birth (years), mean (SD)	28.11 (4.47)	27.78 (4.68)	27.80 (4.67)	0.67	12/644 (1.9)
Maternal education level at birth, *N* (%)					
Low level (ISCED 0-2)	10 (41.7)	169 (38.6)	179 (38.7)		
Medium level (ISCED 3-5)	8 (33.3)	188 (42.9)	196 (42.4)	0.59	182/644 (28.3)
High level (ISCED 6-8)	6 (25.0)	81 (18.5)	87 (18.8)		
Maternal ethnicity, *N* (%)					
Caucasian	32 (86.5)	537 (89.4)	569 (89.2)		
Non-Caucasian	5 (13.5)	64 (10.6)	69 (10.8)	0.59	6/644 (0.9)
At 28 years					
*N* (%)	21 (6.7)	293 (93.3)	314 (100.0)		0/314 (0.0)
Child sex, *N* (%)					
Male	8 (38.1)	111 (37.9)	119 (37.9)	0.98	0/314 (0.0)
Female	13 (61.9)	182 (62.1)	195 (62.1)		
Gestational age (weeks), mean (SD)	31.13 (2.68)	30.97 (2.31)	30.98 (2.34)	0.76	0/314 (0.0)
Birth weight (g), mean (SD)	1174.52 (312.88)	1319.82 (302.09)	1310.10 (304.49)	0.03	0/314 (0.0)
Maternal age at birth (years), mean (SD)	28.34 (3.70)	28.16 (4.38)	28.17 (4.34)	0.86	9/314 (2.9)
Maternal education level at birth, *N* (%)					
Low level (ISCED 0–2)	6 (37.5)	86 (37.6)	92 (37.6)		
Medium level (ISCED 3–5)	6 (37.5)	97 (42.4)	103 (42.0)	0.88	69/314 (22.0)
High level (ISCED 6–8)	4 (25.0)	46 (20.1)	50 (20.4)		
Maternal ethnicity, *N* (%)					
Caucasian	18 (90.0)	275 (94.5)	293 (94.2)		
Non-Caucasian	2 (10.0)	16 (5.5)	18 (5.8)	0.40	3/314 (1.0)
At 35 years					
*N* (%)	23 (6.2)	347 (93.8)	370 (100.0)		0/370 (0.0)
Child sex, *N* (%)					
Male	10 (43.5)	151 (43.5)	161 (43.5)	1.00	0/370 (0.0)
Female	13 (56.5)	196 (56.5)	209 (56.5)		
Gestational age (weeks), mean (SD)	31.40 (2.71)	31.02 (2.35)	31.05 (2.37)	0.46	0/370 (0.0)
Birth weight (g), mean (SD)	1216.52 (298.91)	1332.01 (296.11)	1324.83 (297.19)	0.07	0/370 (0.0)
Maternal age at birth (years), mean (SD)	28.73 (3.70)	28.17 (4.14)	28.21 (4.11)	0.55	8/370 (2.2)
Maternal education level at birth, *N* (%)					
Low level (ISCED 0–2)	6 (33.3)	88 (32.2)	94 (32.3)		
Medium level (ISCED 3–5)	6 (33.3)	131 (48.0)	137 (47.1)	0.32	79/370 (21.4)
High level (ISCED 6–8)	6 (33.3)	54 (19.8)	60 (20.6)		
Maternal ethnicity, *N* (%)					
Caucasian	20 (90.9)	326 (95.0)	346 (94.8)		
Non-Caucasian	2 (9.1)	17 (5.0)	19 (5.2)	0.40	5/370 (1.4)

NEC, Necrotizing enterocolitis; ISCED, International Standard Classification of Education

**Table 11:** Baseline characteristics by MB status

	Multiple	Singleton	Total	*p*-value	Missings/*N* (Pct)
At 19 years					
*N* (%)	138 (21.4)	506 (78.6)	644 (100.0)		0/644 (0.0)
Child sex, *N* (%)					
Male	68 (49.3)	226 (44.7)	294 (45.7)	0.34	0/644 (0.0)
Female	70 (50.7)	280 (55.3)	350 (54.3)		
Gestational age (weeks), mean (SD)	30.50 (2.08)	31.15 (2.54)	31.01 (2.46)	0.01	0/644 (0.0)
Birth weight (g), mean (SD)	1328.12 (266.49)	1307.82 (300.93)	1312.17 (293.81)	0.47	0/644 (0.0)
Maternal age at birth (years), mean (SD)	27.61 (3.85)	27.85 (4.87)	27.80 (4.67)	0.60	12/644 (1.9)
Maternal education level at birth, *N* (%)					
Low level (ISCED 0–2)	33 (33.0)	146 (40.3)	179 (38.7)		
Medium level (ISCED 3–5)	47 (47.0)	149 (41.2)	196 (42.4)	0.40	182/644 (28.3)
High level (ISCED 6–8)	20 (20.0)	67 (18.5)	87 (18.8)		
Maternal ethnicity, *N* (%)					
Caucasian	123 (91.1)	446 (88.7)	569 (89.2)		
Non-Caucasian	12 (8.9)	57 (11.3)	69 (10.8)	0.42	6/644 (0.9)
At 28 years					
*N* (%)	64 (20.4)	250 (79.6)	314 (100.0)		0/314 (0.0)
Child sex, *N* (%)					
Male	23 (35.9)	96 (38.4)	119 (37.9)	0.72	0/314 (0.0)
Female	41 (64.1)	154 (61.6)	195 (62.1)		
Gestational age (weeks), mean (SD)	30.74 (2.04)	31.04 (2.40)	30.98 (2.34)	0.36	0/314 (0.0)
Birth weight (g), mean (SD)	1342.58 (274.63)	1301.79 (311.64)	1310.10 (304.49)	0.34	0/314 (0.0)
Maternal age at birth (years), mean (SD)	28.28 (3.86)	28.15 (4.46)	28.17 (4.34)	0.83	9/314 (2.9)
Maternal education level at birth, *N* (%)					
Low level (ISCED 0–2)	14 (28.0)	78 (40.0)	92 (37.6)		
Medium level (ISCED 3–5)	22 (44.0)	81 (41.5)	103 (42.0)	0.19	69/314 (22.0)
High level (ISCED 6–8)	14 (28.0)	36 (18.5)	50 (20.4)		
Maternal ethnicity, *N* (%)					
Caucasian	60 (95.2)	233 (94.0)	293 (94.2)		
Non-Caucasian	3 (4.8)	15 (6.0)	18 (5.8)	0.70	3/314 (1.0)
At 35 years					
*N* (%)	71 (19.2)	299 (80.8)	370 (100.0)		0/370 (0.0)
Child sex, *N* (%)					
Male	35 (49.3)	126 (42.1)	161 (43.5)	0.27	0/370 (0.0)
Female	36 (50.7)	173 (57.9)	209 (56.5)		
Gestational age (weeks), mean (SD)	30.82 (2.02)	31.10 (2.45)	31.05 (2.37)	0.38	0/370 (0.0)
Birth weight (g), mean (SD)	1354.79 (258.86)	1317.72 (305.55)	1324.83 (297.19)	0.35	0/370 (0.0)
Maternal age at birth (years), mean (SD)	28.86 (3.83)	28.05 (4.16)	28.21 (4.11)	0.13	8/370 (2.2)
Maternal education level at birth, *N* (%)					
Low level (ISCED 0–2)	11 (19.3)	83 (35.5)	94 (32.3)		
Medium level (ISCED 3–5)	33 (57.9)	104 (44.4)	137 (47.1)	0.06	79/370 (21.4)
High level (ISCED 6–8)	13 (22.8)	47 (20.1)	60 (20.6)		
Maternal ethnicity, *N* (%)					
Caucasian	66 (95.7)	280 (94.6)	346 (94.8)		
Non-Caucasian	3 (4.3)	16 (5.4)	19 (5.2)	0.72	5/370 (1.4)

MB, Multiple birth; ISCED, International Standard Classification of Education

**Table 12:** Baseline characteristics—presence of at least one risk factor

	No	Yes	Total	*p*-value	Missings/*N* (Pct)
At 19 years					
*N* (%)	387 (60.1)	257 (39.9)	644 (100.0)		0/644 (0.0)
Child sex, *N* (%)					
Male	171 (44.2)	123 (47.9)	294 (45.7)	0.36	0/644 (0.0)
Female	216 (55.8)	134 (52.1)	350 (54.3)		
Gestational age (weeks), mean (SD)	31.57 (2.48)	30.17 (2.19)	31.01 (2.46)	<0.001	0/644 (0.0)
Birth weight (g), mean (SD)	1330.06 (299.40)	1285.22 (283.63)	1312.17 (293.81)	0.06	0/644 (0.0)
Maternal age at birth (years), mean (SD)	27.87 (4.96)	27.68 (4.20)	27.80 (4.67)	0.61	12/644 (1.9)
Maternal education level at birth, *N* (%)					
Low level (ISCED 0–2)	112 (40.9)	67 (35.6)	179 (38.7)		
Medium level (ISCED 3–5)	113 (41.2)	83 (44.1)	196 (42.4)	0.51	182/644 (28.3)
High level (ISCED 6–8)	49 (17.9)	38 (20.2)	87 (18.8)		
Maternal ethnicity, *N* (%)					
Caucasian	345 (89.6)	224 (88.5)	569 (89.2)		
Non-Caucasian	40 (10.4)	29 (11.5)	69 (10.8)	0.67	6/644 (0.9)
At 28 years					
*N* (%)	181 (57.6)	133 (42.4)	314 (100.0)		0/314 (0.0)
Child sex, *N* (%)					
Male	70 (38.7)	49 (36.8)	119 (37.9)	0.74	0/314 (0.0)
Female	111 (61.3)	84 (63.2)	195 (62.1)		
Gestational age (weeks), mean (SD)	31.44 (2.29)	30.35 (2.26)	30.98 (2.34)	<0.001	0/314 (0.0)
Birth weight (g), mean (SD)	1335.20 (312.27)	1275.94 (291.25)	1310.10 (304.49)	0.09	0/314 (0.0)
Maternal age at birth (years), mean (SD)	28.17 (4.43)	28.18 (4.22)	28.17 (4.34)	0.98	9/314 (2.9)
Maternal education level at birth, *N* (%)					
Low level (ISCED 0–2)	57 (41.3)	35 (32.7)	92 (37.6)		
Medium level (ISCED 3–5)	56 (40.6)	47 (43.9)	103 (42.0)	0.34	69/314 (22.0)
High level (ISCED 6–8)	25 (18.1)	25 (23.4)	50 (20.4)		
Maternal ethnicity, *N* (%)					
Caucasian	169 (93.9)	124 (94.7)	293 (94.2)		
Non-Caucasian	11 (6.1)	7 (5.3)	18 (5.8)	0.77	3/314 (1.0)
At 35 years					
*N* (%)	217 (58.6)	153 (41.4)	370 (100.0)		0/370 (0.0)
Child sex, *N* (%)					
Male	91 (41.9)	70 (45.8)	161 (43.5)	0.47	0/370 (0.0)
Female	126 (58.1)	83 (54.2)	209 (56.5)		
Gestational age (weeks), mean (SD)	31.59 (2.31)	30.28 (2.26)	31.05 (2.37)	<0.001	0/370 (0.0)
Birth weight (g), mean (SD)	1348.67 (306.19)	1291.01 (281.47)	1324.83 (297.19)	0.07	0/370 (0.0)
Maternal age at birth (years), mean (SD)	28.30 (4.02)	28.08 (4.24)	28.21 (4.11)	0.61	8/370 (2.2)
Maternal education level at birth, *N* (%)					
Low level (ISCED 0–2)	60 (36.1)	34 (27.2)	94 (32.3)		
Medium level (ISCED 3–5)	73 (44.0)	64 (51.2)	137 (47.1)	0.26	79/370 (21.4)
High level (ISCED 6–8)	33 (19.9)	27 (21.6)	60 (20.6)		
Maternal ethnicity, *N* (%)					
Caucasian	205 (95.3)	141 (94.0)	346 (94.8)		
Non-Caucasian	10 (4.7)	9 (6.0)	19 (5.2)	0.57	5/370 (1.4)

ISCED, International Standard Classification of Education

## Appendix 2

See Tables 13, 14, 15, 16, 17 and 18.

**Table 13:** HRQoL outcomes by BPD

	BPD	No-BPD	Total	*p*-value
At 19 years				
*N* (%)	35 (5.4)	609 (94.6)	644 (100.0)	
HUI3-vision optimal functioning, *N* (%)	0 (0.0)	1 (0.2)	1 (0.2)	0.81
HUI3-hearing optimal functioning, *N* (%)	0 (0.0)	7 (1.1)	7 (1.1)	0.52
HUI3-speech optimal functioning, *N* (%)	31 (88.6)	507 (83.3)	538 (83.5)	0.41
HUI3-emotion optimal functioning, *N* (%)	28 (80.0)	396 (65.0)	424 (65.8)	0.07
HUI3-pain optimal functioning, *N* (%)	26 (74.3)	457 (75.0)	483 (75.0)	0.92
HUI3-ambulation optimal functioning, *N* (%)	0 (0.0)	4 (0.7)	4 (0.6)	0.63
HUI3-dexterity optimal functioning, *N* (%)	32 (91.4)	586 (96.2)	618 (96.0)	0.16
HUI3-cognition optimal functioning, *N* (%)	3 (8.6)	30 (4.9)	33 (5.1)	0.34
HUI3 MAU score, mean (SD)	0.89 (0.16)	0.87 (0.18)	0.87 (0.18)	0.55
HUI3 MAU score, median (25th; 75th)	0.93 (0.84; 1.00)	0.93 (0.83; 1.00)	0.93 (0.83; 1.00)	0.63
At 28 years				
*N* (%)	17 (5.4)	297 (94.6)	314 (100.0)	
HUI3-vision optimal functioning, *N* (%)	0 (0.0)	2 (0.7)	2 (0.6)	0.73
HUI3-hearing optimal functioning, *N* (%)	0 (0.0)	1 (0.3)	1 (0.3)	0.81
HUI3-speech optimal functioning, *N* (%)	14 (82.4)	267 (89.9)	281 (89.5)	0.32
HUI3-emotion optimal functioning, *N* (%)	0 (0.0)	1 (0.3)	1 (0.3)	0.81
HUI3-pain optimal functioning, *N* (%)	13 (76.5)	219 (73.7)	232 (73.9)	0.80
HUI3-ambulation optimal functioning, *N* (%)	0 (0.0)	2 (0.7)	2 (0.6)	0.73
HUI3-dexterity optimal functioning, *N* (%)	16 (94.1)	287 (96.6)	303 (96.5)	0.58
HUI3-cognition optimal functioning, *N* (%)	3 (17.6)	22 (7.4)	25 (8.0)	0.13
HUI3 MAU score, mean (SD)	0.86 (0.21)	0.89 (0.16)	0.89 (0.17)	0.54
HUI3 MAU score, median (25th; 75th)	0.97 (0.83; 0.97)	0.95 (0.85; 1.00)	0.96 (0.85; 1.00)	0.72
At 35 years				
*N* (%)	19 (5.1)	351 (94.9)	370 (100.0)	
SF-6D physical optimal functioning, *N* (%)	6 (31.6)	47 (13.4)	53 (14.3)	0.03
SF-6D role limitations optimal, *N* (%)	11 (57.9)	249 (70.9)	260 (70.3)	0.23
SF-6D social functioning optimal, *N* (%)	5 (26.3)	79 (22.5)	84 (22.7)	0.70
SF-6D pain optimal level, *N* (%)	4 (21.1)	85 (24.2)	89 (24.1)	0.75
SF-6D mental health optimal level, *N* (%)	2 (10.5)	129 (36.8)	131 (35.4)	0.02
SF-6D vitality optimal level, *N* (%)	2 (10.5)	28 (8.0)	30 (8.1)	0.69
SF-6D (utility score), mean (SD)	0.78 (0.12)	0.81 (0.11)	0.81 (0.12)	0.36
SF-6D (utility score), median (25th; 75th)	0.80 (0.66; 0.92 )	0.86 (0.72; 0.86)	0.86 (0.72; 1.00)	0.40

**Table 14:** HRQoL outcomes by IVH

	IVH	No-IVH	Total	*p*-value
At 19 years				
*N* (%)	109 (16.9)	535 (83.1)	644 (100.0)	
HUI3-vision optimal functioning, *N* (%)	0 (0.0)	1 (0.2)	1 (0.2)	0.65
HUI3-hearing optimal functioning, *N* (%)	0 (0.0)	7 (1.3)	7 (1.1)	0.23
HUI3-speech optimal functioning, *N* (%)	84 (77.1)	454 (84.9)	538 (83.5)	0.05
HUI3-emotion optimal functioning, *N* (%)	69 (63.3)	355 (66.4)	424 (65.8)	0.54
HUI3-pain optimal functioning, *N* (%)	77 (70.6)	406 (75.9)	483 (75.0)	0.25
HUI3-ambulation optimal functioning, *N* (%)	2 (1.8)	2 (0.4)	4 (0.6)	0.08
HUI3-dexterity optimal functioning, *N* (%)	100 (91.7)	518 (96.8)	618 (96.0)	0.01
HUI3-cognition optimal functioning, *N* (%)	8 (7.3)	25 (4.7)	33 (5.1)	0.25
HUI3 MAU score, mean (SD)	0.86 (0.18)	0.87 (0.18)	0.87 (0.18)	0.40
HUI3 MAU score, median (25th; 75th)	0.92 (0.79; 0.97)	0.93 (0.83; 1.00)	0.93 (0.83; 1.00)	0.17
At 28 years				
*N* (%)	59 (18.8)	255 (81.2)	314 (100.0)	
HUI3-vision optimal functioning, *N* (%)	0 (0.0)	2 (0.8)	2 (0.6)	0.49
HUI3-hearing optimal functioning, *N* (%)	1 (1.7)	0 (0.0)	1 (0.3)	0.04
HUI3-speech optimal functioning, *N* (%)	50 (84.7)	231 (90.6)	281 (89.5)	0.19
HUI3-emotion optimal functioning, *N* (%)	0 (0.0)	1 (0.4)	1 (0.3)	0.63
HUI3-pain optimal functioning, *N* (%)	45 (76.3)	187 (73.3)	232 (73.9)	0.64
HUI3-ambulation optimal functioning, *N* (%)	1 (1.7)	1 (0.4)	2 (0.6)	0.26
HUI3-dexterity optimal functioning, *N* (%)	56 (94.9)	247 (96.9)	303 (96.5)	0.46
HUI3-cognition optimal functioning, *N* (%)	7 (11.9)	18 (7.1)	25 (8.0)	0.22
HUI3 MAU score, mean (SD)	0.87 (0.19)	0.89 (0.16)	0.89 (0.17)	0.31
HUI3 MAU score, median (25th; 75th)	0.93 (0.78; 1.00)	0.97 (0.86; 1.00)	0.96 (0.85; 1.00)	0.47
At 35 years				
*N* (%)	69 (18.6)	301 (81.4)	370 (100.0)	
SF-6D physical optimal functioning, *N* (%)	14 (20.3)	39 (13.0)	53 (14.3)	0.12
SF-6D role limitations optimal, *N* (%)	50 (72.5)	210 (69.8)	260 (70.3)	0.66
SF-6D social functioning optimal, *N* (%)	18 (26.1)	66 (21.9)	84 (22.7)	0.46
SF-6D pain optimal level, *N* (%)	18 (26.1)	71 (23.6)	89 (24.1)	0.66
SF-6D mental health optimal level, *N* (%)	23 (33.3)	108 (35.9)	131 (35.4)	0.69
SF-6D vitality optimal level, *N* (%)	9 (13.0)	21 (7.0)	30 (8.1)	0.10
SF-6D (utility score), mean (SD)	0.79 (0.12)	0.81 (0.12)	0.81 (0.12)	0.24
SF-6D (utility score), median (25th; 75th)	0.80 (0.47; 1.00)	0.86 (0.52; 1.00)	0.86 (0.47; 1.00)	0.21

**Table 15:** HRQoL outcomes by grades 3 and 4 IVH status

	No IVH or IVH grade 1/2	IVH Grade 3/4 1	Total	*p*-value
At 19 years				
*N* (%)	455 (94.4)	27 (5.6)	482 (100.0)	
HUI3-vision optimal functioning, *N* (%)	1 (0.2)	0 (0.0)	1 (0.2)	0.81
HUI3-hearing optimal functioning, *N* (%)	6 (1.3)	0 (0.0)	6 (1.2)	0.55
HUI3-speech optimal functioning, *N* (%)	381 (83.7)	21 (77.8)	402 (83.4)	0.42
HUI3-emotion optimal functioning, *N* (%)	293 (64.4)	15 (55.6)	308 (63.9)	0.35
HUI3-pain optimal functioning, *N* (%)	352 (77.4)	14 (51.9)	366 (75.9)	<0.001
HUI3-ambulation optimal functioning, *N* (%)	1 (0.2)	1 (3.7)	2 (0.4)	0.01
HUI3-dexterity optimal functioning, *N* (%)	442 (97.1)	23 (85.2)	465 (96.5)	<0.001
HUI3-cognition optimal functioning, *N* (%)	26 (5.7)	3 (11.1)	29 (6.0)	0.25
HUI3 MAU score, mean (SD)	0.87 (0.18)	0.80 (0.19)	0.87 (0.18)	0.04
HUI3 MAU score, median (25th; 75th)	0.93 (0.83; 1.00)	0.87 (0.73; 0.92)	0.93 (0.82; 0.97)	<0.001
At 28 years				
*N* (%)	217 (92.7)	17 (7.3)	234 (100.0)	
HUI3-vision optimal functioning, *N* (%)	2 (0.9)	0 (0.0)	2 (0.9)	0.69
HUI3-hearing optimal functioning, *N* (%)				
HUI3-speech optimal functioning, *N* (%)	196 (90.3)	14 (82.4)	210 (89.7)	0.30
HUI3-emotion optimal functioning, *N* (%)				
HUI3-pain optimal functioning, *N* (%)	161 (74.2)	11 (64.7)	172 (73.5)	0.39
HUI3-ambulation optimal functioning, *N* (%)				
HUI3-dexterity optimal functioning, *N* (%)	210 (96.8)	16 (94.1)	226 (96.6)	0.56
HUI3-cognition optimal functioning, *N* (%)	15 (6.9)	2 (11.8)	17 (7.3)	0.46
HUI3 MAU score, mean (SD)	0.89 (0.15)	0.80 (0.22)	0.88 (0.16)	0.02
HUI3 MAU score, median (25th; 75th)	0.95 (0.87; 1.00)	0.88 (0.70; 0.97)	0.95 (0.84; 0.97)	0.05
At 35 years				
*N* (%)	251 (94.4)	15 (5.6)	266 (100.0)	
SF-6D physical optimal functioning, *N* (%)	33 (13.1)	9 (60.0)	42 (15.8)	<0.001
SF-6D role limitations optimal, *N* (%)	184 (73.3)	9 (60.0)	193 (72.6)	0.26
SF-6D social functioning optimal, *N* (%)	56 (22.3)	4 (26.7)	60 (22.6)	0.69
SF-6D pain optimal level, *N* (%)	58 (23.1)	6 (40.0)	64 (24.1)	0.14
SF-6D mental health optimal level, *N* (%)	88 (35.1)	4 (26.7)	92 (34.6)	0.51
SF-6D vitality optimal level, *N* (%)	20 (8.0)	2 (13.3)	22 (8.3)	0.46
SF-6D (utility score), mean (SD)	0.81 (0.11)	0.75 (0.10)	0.81 (0.11)	0.03
SF-6D (utility score), median (25th; 75th)	0.86 (0.72; 0.90)	0.80 (0.66; 0.80)	0.86 (0.67; 0.86)	0.02

**Table 16:** HRQoL outcomes by NEC

	NEC	No-NEC	Total	*p*-value
At 19 years				
*N* (%)	39 (6.1)	605 (93.9)	644 (100.0)	
HUI3-vision optimal functioning, *N* (%)	0 (0.0)	1 (0.2)	1 (0.2)	0.80
HUI3-hearing optimal functioning, *N* (%)	1 (2.6)	6 (1.0)	7 (1.1)	0.36
HUI3-speech optimal functioning, *N* (%)	33 (84.6)	505 (83.5)	538 (83.5)	0.85
HUI3-emotion optimal functioning, *N* (%)	22 (56.4)	402 (66.4)	424 (65.8)	0.20
HUI3-pain optimal functioning, *N* (%)	33 (84.6)	450 (74.4)	483 (75.0)	0.15
HUI3-ambulation optimal functioning, *N* (%)	0 (0.0)	4 (0.7)	4 (0.6)	0.61
HUI3-dexterity optimal functioning, *N* (%)	38 (97.4)	580 (95.9)	618 (96.0)	0.63
HUI3-cognition optimal functioning, *N* (%)	4 (10.3)	29 (4.8)	33 (5.1)	0.13
HUI3 MAU score, mean (SD)	0.87 (0.15)	0.87 (0.18)	0.87 (0.18)	0.84
HUI3 MAU score, median (25th; 75th)	0.93 (0.80; 1.00)	0.93 (0.84; 1.00)	0.93 (0.83; 1.00)	0.83
At 28 years				
*N* (%)	21 (6.7)	293 (93.3)	314 (100.0)	
HUI3-vision optimal functioning, *N* (%)	0 (0.0)	2 (0.7)	2 (0.6)	0.70
HUI3-hearing optimal functioning28y, *N* (%)	0 (0.0)	1 (0.3)	1 (0.3)	0.79
HUI3-speech optimal functioning, *N* (%)	21 (100.0)	260 (88.7)	281 (89.5)	0.10
HUI3-emotion optimal functioning, *N* (%)	1 (4.8)	0 (0.0)	1 (0.3)	<0.001
HUI3-pain optimal functioning, *N* (%)	16 (76.2)	216 (73.7)	232 (73.9)	0.80
HUI3-ambulation optimal functioning, *N* (%)	0 (0.0)	2 (0.7)	2 (0.6)	0.70
HUI3-dexterity optimal functioning, *N* (%)	21 (100.0)	282 (96.2)	303 (96.5)	0.37
HUI3-cognition optimal functioning, *N* (%)	2 (9.5)	23 (7.8)	25 (8.0)	0.78
HUI3 MAU score, mean (SD)	0.89 (0.16)	0.88 (0.17)	0.89 (0.17)	0.81
HUI3 MAU score, median (25th; 75th)	0.95 (0.89; 0.97)	0.97 (0.85; 1.00)	0.96 (0.85; 1.00)	0.95
At 35 years				
*N* (%)	23 (6.2)	347 (93.8)	370 (100.0)	
SF-6D physical optimal functioning, *N* (%)	4 (17.4)	49 (14.1)	53 (14.3)	0.66
SF-6D role limitations optimal, *N* (%)	14 (60.9)	246 (70.9)	260 (70.3)	0.31
SF-6D social functioning optimal, *N* (%)	3 (13.0)	81 (23.3)	84 (22.7)	0.25
SF-6D pain optimal level, *N* (%)	4 (17.4)	85 (24.5)	89 (24.1)	0.44
SF-6D mental health optimal level, *N* (%)	5 (21.7)	126 (36.3)	131 (35.4)	0.16
SF-6D vitality optimal level, *N* (%)	5 (21.7)	25 (7.2)	30 (8.1)	0.01
SF-6D (utility score), mean (SD)	0.76 (0.16)	0.81 (0.11)	0.81 (0.12)	0.05
SF-6D (utility score), median (25th; 75th)	0.80 (0.60; 0.90)	0.86 (0.72; 0.86)	0.86 (0.72; 0.86)	0.22

**Table 17:** HRQoL outcomes by multiple birth (MB)

	MB	No-MB	Total	*p*-value
At 19 years				
*N* (%)	138 (21.4)	506 (78.6)	644 (100.0)	
HUI3-vision optimal functioning, *N* (%)	1 (0.7)	0 (0.0)	1 (0.2)	0.06
HUI3-hearing optimal functioning, *N* (%)	1 (0.7)	6 (1.2)	7 (1.1)	0.64
HUI3-speech optimal functioning, *N* (%)	123 (89.1)	415 (82.0)	538 (83.5)	0.05
HUI3-emotion optimal functioning, *N* (%)	95 (68.8)	329 (65.0)	424 (65.8)	0.40
HUI3-pain optimal functioning, *N* (%)	106 (76.8)	377 (74.5)	483 (75.0)	0.58
HUI3-ambulation optimal functioning, *N* (%)	3 (2.2)	1 (0.2)	4 (0.6)	0.01
HUI3-dexterity optimal functioning, *N* (%)	130 (94.2)	488 (96.4)	618 (96.0)	0.24
HUI3-cognition optimal functioning, *N* (%)	12 (8.7)	21 (4.2)	33 (5.1)	0.03
HUI3 MAU score, mean (SD)	0.87 (0.20)	0.87 (0.17)	0.87 (0.18)	0.83
HUI3 MAU score, median (25th; 75th)	0.93 (0.83; 1.00)	0.93 (0.83; 1.00)	0.93 (0.83; 1.00)	0.51
At 28 years				
*N* (%)	64 (20.4)	250 (79.6)	314 (100.0)	
HUI3-vision optimal functioning, *N* (%)	0 (0.0)	2 (0.8)	2 (0.6)	0.47
HUI3-hearing optimal functioning, *N* (%)	0 (0.0)	1 (0.4)	1 (0.3)	0.61
HUI3-speech optimal functioning, *N* (%)	59 (92.2)	222 (88.8)	281 (89.5)	0.43
HUI3-emotion optimal functioning, *N* (%)	0 (0.0)	1 (0.4)	1 (0.3)	0.61
HUI3-pain optimal functioning, *N* (%)	45 (70.3)	187 (74.8)	232 (73.9)	0.47
HUI3-ambulation optimal functioning, *N* (%)	1 (1.6)	1 (0.4)	2 (0.6)	0.30
HUI3-dexterity optimal functioning, *N* (%)	60 (93.8)	243 (97.2)	303 (96.5)	0.18
HUI3-cognition optimal functioning, *N* (%)	6 (9.4)	19 (7.6)	25 (8.0)	0.64
HUI3 MAU score, mean (SD)	0.88 (0.18)	0.89 (0.16)	0.89 (0.17)	0.69
HUI3 MAU score, median (25th; 75th)	0.97 (0.86; 0.97)	0.95 (0.85; 1.00)	0.96 (0.85; 1.00)	0.67
At 35 years				
*N* (%)	71 (19.2)	299 (80.8)	370 (100.0)	
SF-6D physical optimal functioning, *N* (%)	8 (11.3)	45 (15.1)	53 (14.3)	0.41
SF-6D role limitations optimal, *N* (%)	50 (70.4)	210 (70.2)	260 (70.3)	0.98
SF-6D social functioning optimal, *N* (%)	19 (26.8)	65 (21.7)	84 (22.7)	0.36
SF-6D pain optimal level, *N* (%)	17 (23.9)	72 (24.1)	89 (24.1)	0.98
SF-6D mental health optimal level, *N* (%)	28 (39.4)	103 (34.4)	131 (35.4)	0.43
SF-6D vitality optimal level, *N* (%)	4 (5.6)	26 (8.7)	30 (8.1)	0.40
SF-6D (utility score), mean (SD)	0.79 (0.11)	0.81 (0.12)	0.81 (0.12)	0.31
SF-6D (utility score), median (25th; 75th)	0.80 (0.66; 0.86)	0.86 (0.72; 0.86)	0.86 (0.72; 0.86)	0.25

**Table 18:** QoL by any risk factor

	No	Yes	Total	*p*-value
At 19 years				
*N* (%)	387 (60.1)	257 (39.9)	644 (100.0)	
HUI3-vision optimal functioning, *N* (%)	0 (0.0)	1 (0.4)	1 (0.2)	0.22
HUI3-hearing optimal functioning, *N* (%)	5 (1.3)	2 (0.8)	7 (1.1)	0.54
HUI3-speech optimal functioning, *N* (%)	323 (83.5)	215 (83.7)	538 (83.5)	0.95
HUI3-emotion optimal functioning, *N* (%)	255 (65.9)	169 (65.8)	424 (65.8)	0.97
HUI3-pain optimal functioning, *N* (%)	289 (74.7)	194 (75.5)	483 (75.0)	0.82
HUI3-ambulation optimal functioning, *N* (%)	0 (0.0)	4 (1.6)	4 (0.6)	0.01
HUI3-dexterity optimal functioning, *N* (%)	379 (97.9)	239 (93.0)	618 (96.0)	<0.001
HUI3-cognition optimal functioning, *N* (%)	14 (3.6)	19 (7.4)	33 (5.1)	0.03
HUI3 MAU score, mean (SD)	0.87 (0.17)	0.86 (0.19)	0.87 (0.18)	0.58
HUI3 MAU score, median (25th; 75th)	0.93 (0.84; 1.00)	0.93 (0.83; 1.00)	0.93 (0.83; 1.00)	0.69
At 28 years				
*N* (%)	181 (57.6)	133 (42.4)	314 (100.0)	
HUI3-vision optimal functioning, *N* (%)	2 (1.1)	0 (0.0)	2 (0.6)	0.22
HUI3-hearing optimal functioning, *N* (%)	0 (0.0)	1 (0.8)	1 (0.3)	0.24
HUI3-speech optimal functioning, *N* (%)	161 (89.0)	120 (90.2)	281 (89.5)	0.72
HUI3-emotion optimal functioning, *N* (%)	0 (0.0)	1 (0.8)	1 (0.3)	0.24
HUI3-pain optimal functioning, *N* (%)	133 (73.5)	99 (74.4)	232 (73.9)	0.85
HUI3-ambulation optimal functioning, *N* (%)	1 (0.6)	1 (0.8)	2 (0.6)	0.83
HUI3-dexterity optimal functioning, *N* (%)	177 (97.8)	126 (94.7)	303 (96.5)	0.15
HUI3-cognition optimal functioning, *N* (%)	11 (6.1)	14 (10.5)	25 (8.0)	0.15
HUI3 MAU score, mean (SD)	0.89 (0.16)	0.88 (0.18)	0.89 (0.17)	0.42
HUI3 MAU score, median (25th; 75th)	0.97 (0.86; 1.00)	0.95 (0.85; 0.97)	0.96 (0.85; 0.97)	0.42
At 35 years				
*N* (%)	217 (58.6)	153 (41.4)	370 (100.0)	
SF-6D physical optimal functioning, *N* (%)	27 (12.4)	26 (17.0)	53 (14.3)	0.22
SF-6D role limitations optimal, *N* (%)	153 (70.5)	107 (69.9)	260 (70.3)	0.91
SF-6D social functioning optimal, *N* (%)	49 (22.6)	35 (22.9)	84 (22.7)	0.95
SF-6D pain optimal level, *N* (%)	52 (24.0)	37 (24.2)	89 (24.1)	0.96
SF-6D mental health optimal level, *N* (%)	81 (37.3)	50 (32.7)	131 (35.4)	0.36
SF-6D vitality optimal level, *N* (%)	14 (6.5)	16 (10.5)	30 (8.1)	0.16
SF-6D (utility score), mean (SD)	0.82 (0.11)	0.79 (0.12)	0.81 (0.12)	0.04
SF-6D (utility score), median (25th; 75th)	0.86 (0.73; 0.86)	0.80 (0.66; 0.86)	0.86 (0.66; 0.86)	0.06

## Appendix 3

See Tables 19, 20, 21, 22 and 23.

**Table 19:** Adjusted BPD Impact on HRQoL

Outcome	$\beta _{BPD}$	SE	Lower 95% CI	Upper 95% CI	*p*-value
At 19 years					
HUI3-MAU score	0.001	0.031	− 0.061	0.063	0.976
HUI3-vision OF	− 0.002	0.009	− 0.020	0.016	0.832
HUI3-hearing OF	− 0.010	0.020	− 0.050	0.029	0.605
HUI3-speech OF	0.002	0.064	− 0.124	0.129	0.970
HUI3-emotion OF	0.100	0.089	− 0.075	0.275	0.263
HUI3-pain OF	− 0.074	0.079	− 0.229	0.081	0.350
HUI3-ambulation OF	− 0.008	0.016	− 0.038	0.023	0.630
HUI3-dexterity OF	− 0.063	0.037	− 0.135	0.009	0.087
HUI3-cognition OF	− 0.003	0.037	− 0.075	0.069	0.938
At 28 years					
HUI3-MAU score	− 0.033	0.055	− 0.142	0.076	0.554
HUI3-speech OF	− 0.145	0.130	− 0.402	0.112	0.268
HUI3-pain OF	− 0.004	0.139	− 0.278	0.271	0.979
HUI3-dexterity OF	− 0.033	0.047	− 0.126	0.059	0.476
HUI3-cognition OF	0.178	0.111	− 0.041	0.397	0.110
At 35 years					
SF-6D MAU score	− 0.050	0.045	− 0.139	0.040	0.276
SF-6D physical OF	0.213	0.144	− 0.071	0.497	0.140
SF-6D role limitations OF	− 0.183	0.165	− 0.509	0.143	0.269
SF-6D social OF	0.028	0.112	− 0.193	0.249	0.803
SF-6D pain OF	0.040	0.117	− 0.191	0.270	0.734
SF-6D mental health OF	− 0.171	0.104	− 0.376	0.034	0.102
SF-6D vitality OF	0.040	0.082	− 0.122	0.202	0.623

MAU stands for multi-attribute utility. OF: Optimal Functioning. Vision, Hearing, Emotion, and Ambulation OF at 28 years were omitted from the model due to no variation and are not shown. Results at 28 and 35 are adjusted for selective attrition using inverse probability weighting

**Table 20:** Adjusted IVH impact on HRQoL

	$\beta _{IVH}$	SE	Lower 95% CI	Upper 95% CI	*p*-value
HUI3 outcomes at 19 years					
HUI3-MAU scores	0.01	0.02	− 0.04	0.05	0.81
HUI3-vision OF	0.00	0.01	− 0.01	0.01	0.64
HUI3-hearing OF	0.01	0.01	− 0.01	0.04	0.32
HUI3-speech OF	0.06	0.04	− 0.02	0.15	0.16
HUI3-emotion OF	0.03	0.06	− 0.09	0.15	0.63
HUI3-pain OF	0.09	0.05	− 0.01	0.19	0.09
HUI3-ambulation OF	− 0.01	0.01	− 0.03	0.01	0.48
HUI3-dexterity OF	0.04	0.02	− 0.01	0.08	0.14
HUI3-cognition OF	− 0.03	0.02	− 0.08	0.01	0.16
HUI3 outcomes at 28 years					
HUI3-MAU scores	0.03	0.03	− 0.03	0.10	0.34
HUI3-hearing OF	− 0.04	0.04	− 0.11	0.03	0.31
HUI3-speech OF	0.09	0.06	− 0.04	0.22	0.17
HUI3-pain OF	− 0.01	0.07	− 0.15	0.14	0.92
HUI3-ambulation OF	0.01	0.01	− 0.01	0.03	0.33
HUI3-dexterity OF	0.04	0.05	− 0.06	0.14	0.44
HUI3-cognition OF	− 0.06	0.06	− 0.18	0.06	0.34
SF-6D outcomes at 35 years					
SF-6D MAU scores	− 0.01	0.02	− 0.04	0.03	0.73
SF-6D physical OF	− 0.05	0.06	− 0.16	0.06	0.40
SF-6D role limitations OF	− 0.12	0.07	− 0.25	0.02	0.09
SF-6D social OF	− 0.04	0.06	− 0.16	0.09	0.57
SF-6D pain OF	− 0.02	0.06	− 0.13	0.10	0.80
SF-6D mental health OF	0.00	0.08	− 0.15	0.16	0.96
SF-6D vitality OF	− 0.04	0.05	− 0.13	0.05	0.40

MAU stands for multi-attribute utility. OF: Optimal Functioning. Results at 28 and 35 are adjusted for selective attrition inverse probability weighting. Variables included in the weighting model were: sex, GA, birth weight, maternal age at birth, maternal education, maternal ethnicity and maternal marital status

**Table 21:** Adjusted impact of grade 3/4 IVH on HRQoL

	$\beta _{IVH_{3/4}}$	SE	Lower 95% CI	Upper 95% CI	*p*-value
HUI3 outcomes at 19 years					
HUI3-MAU scores	− 0.08	0.04	− 0.16	− 0.00	0.05
HUI3-vision OF	− 0.00	0.01	− 0.03	0.02	0.75
HUI3-hearing OF	− 0.02	0.03	− 0.07	0.04	0.57
HUI3-speech OF	− 0.08	0.08	− 0.24	0.08	0.33
HUI3-emotion OF	− 0.17	0.11	− 0.40	0.05	0.12
HUI3-pain OF	− 0.37	0.10	− 0.56	− 0.18	<0.001
HUI3-ambulation OF	0.05	0.02	0.01	0.08	0.01
HUI3-dexterity OF	− 0.12	0.05	− 0.21	− 0.03	0.01
HUI3-cognition OF	0.00	0.05	− 0.09	0.10	0.98
HUI3 outcomes at 28 years					
HUI3-MAU scores	− 0.15	0.07	− 0.28	− 0.02	0.03
HUI3-speech OF	− 0.14	0.11	− 0.37	0.08	0.20
HUI3-pain OF	− 0.18	0.14	− 0.46	0.10	0.20
HUI3-dexterity OF	− 0.09	0.09	− 0.28	0.09	0.33
HUI3-cognition OF	0.13	0.10	− 0.07	0.33	0.20
SF-6D outcomes at 35 years					
SF-6D MAU scores	− 0.07	0.03	− 0.14	− 0.01	0.03
SF-6D physical OF	0.49	0.15	0.20	0.77	<0.001
SF-6D role limitations OF	− 0.19	0.16	− 0.50	0.11	0.21
SF-6D social OF	0.05	0.11	− 0.17	0.27	0.66
SF-6D pain OF	0.19	0.14	− 0.07	0.46	0.16
SF-6D mental health OF	− 0.03	0.15	− 0.32	0.26	0.83
SF-6D vitality OF	0.12	0.13	− 0.13	0.37	0.34

MAU stands for multi-attribute utility. OF: Optimal Functioning. Results at 28 and 35 are adjusted for selective attrition inverse probability weighting. Variables included in the weighting model were: sex, GA, birth weight, maternal age at birth, maternal education, maternal ethnicity and maternal marital status

**Table 22:** Adjusted NEC impact on HRQoL

Outcome	$\beta _{NEC}$	SE	Lower 95% CI	Upper 95% CI	*p*-value
At 19 years					
HUI3-MAU score	0.004	0.037	− 0.069	0.076	0.922
HUI3-vision OF	− 0.003	0.011	− 0.024	0.018	0.796
HUI3-hearing OF	0.039	0.023	− 0.007	0.085	0.099
HUI3-speech OF	− 0.012	0.075	− 0.160	0.135	0.872
HUI3-emotion OF	− 0.105	0.104	− 0.310	0.100	0.313
HUI3-pain OF	0.030	0.092	− 0.151	0.211	0.746
HUI3-ambulation OF	− 0.006	0.018	− 0.042	0.030	0.744
HUI3-dexterity OF	0.040	0.043	− 0.044	0.124	0.351
HUI3-cognition OF	0.012	0.043	− 0.072	0.096	0.776
At 28 years					
HUI3-MAU score	0.032	0.042	− 0.052	0.115	0.454
HUI3-speech OF	0.084	0.033	0.033	0.136	<0.001
HUI3-pain OF	− 0.055	0.148	− 0.348	0.237	0.709
HUI3-dexterity OF	0.045	0.028	− 0.011	0.100	0.113
HUI3-cognition OF	0.030	0.063	− 0.095	0.155	0.641
At 35 years					
SF-6D MAU score	− 0.032	0.048	− 0.127	0.063	0.511
SF-6D physical OF	0.077	0.117	− 0.154	0.308	0.510
SF-6D role limitations OF	− 0.157	0.156	− 0.464	0.149	0.313
SF-6D social OF	− 0.141	0.073	− 0.285	0.004	0.056
SF-6D pain OF	0.113	0.130	− 0.144	0.370	0.385
SF-6D mental health OF	− 0.228	0.100	− 0.425	− 0.031	0.023
SF-6D vitality OF	0.151	0.115	− 0.075	0.377	0.188

MAU stands for multi-attribute utility. OF: Optimal Functioning. Vision, Hearing, Emotion, and Ambulation OF at 28 years were omitted from the model due to no variation and are not shown. Results at 28 and 35 are adjusted for selective attrition using inverse probability weighting

**Table 23:** Adjusted MB impact on HRQoL

	$\beta _{MB}$	SE	Lower 95% CI	Upper 95% CI	*p*-value
HUI3 outcomes at 19 years					
HUI3-MAU scores	0.00	0.02	− 0.03	0.04	0.83
HUI3-vision OF	− 0.01	0.01	− 0.02	0.00	0.06
HUI3-hearing OF	0.01	0.01	− 0.01	0.04	0.25
HUI3-speech OF	− 0.05	0.04	− 0.13	0.02	0.17
HUI3-emotion OF	− 0.06	0.05	− 0.16	0.05	0.28
HUI3-pain OF	0.01	0.05	− 0.09	0.10	0.89
HUI3-ambulation OF	− 0.02	0.01	− 0.04	0.00	0.06
HUI3-dexterity OF	0.01	0.02	− 0.04	0.05	0.78
HUI3-cognition OF	− 0.04	0.02	− 0.09	− 0.00	0.05
HUI3 outcomes at 28 years					
HUI3-MAU scores	− 0.01	0.03	− 0.06	0.04	0.82
HUI3-hearing OF	0.01	0.01	− 0.01	0.02	0.34
HUI3-speech OF	− 0.00	0.05	− 0.10	0.10	0.96
HUI3-pain OF	0.04	0.08	− 0.11	0.19	0.57
HUI3-ambulation OF	0.01	0.01	− 0.01	0.02	0.34
HUI3-dexterity OF	0.01	0.03	− 0.05	0.07	0.80
HUI3-cognition OF	− 0.04	0.05	− 0.14	0.06	0.45
SF-6D outcomes at 35 years					
SF-6D MAU scores	0.03	0.02	− 0.01	0.06	0.16
SF-6D physical OF	0.06	0.04	− 0.03	0.14	0.20
SF-6D role limitations OF	0.03	0.07	− 0.11	0.18	0.65
SF-6D social OF	− 0.04	0.07	− 0.18	0.09	0.54
SF-6D pain OF	0.04	0.06	− 0.07	0.16	0.44
SF-6D mental health OF	0.02	0.07	− 0.12	0.16	0.81
SF-6D vitality OF	− 0.00	0.05	− 0.11	0.10	0.96

MAU stands for multi-attribute utility. OF: Optimal Functioning. Results at 28 and 35 are adjusted for selective attrition inverse probability weighting. Variables included in the weighting model were: sex, GA, birth weight, maternal age at birth, maternal education, maternal ethnicity and maternal marital status

## Appendix 4

See Tables 24, 25, 26, 27 and Fig. 1.

**Table 24:** Analytic sample derivation by exposure and time point

Time	Exposure	Sample size				Exclusions		
		Total	Outcome	+ Exposure	Final	Outcome	Exposure	Covars
19-year follow-up (HUI-3)								
19 y	BPD	1336	644	644	448	692	0	196
19 y	IVH Grade 3–4	1336	644	482	331	692	162	151
19 y	NEC	1336	644	644	448	692	0	196
28-year follow-up (HUI-3)								
28 y	BPD	1336	314	314	237	1022	0	77
28 y	IVH Grade 3–4	1336	314	234	175	1022	80	59
28 y	NEC	1336	314	314	237	1022	0	77
35-year follow-up (SF-6D)								
35 y	BPD	1336	370	370	282	966	0	88
35 y	IVH Grade 3–4	1336	370	266	203	966	104	63
35 y	NEC	1336	370	370	282	966	0	88

Sample sizes at each stage of analytic sample derivation. Total = baseline cohort; Outcome = non-missing outcome data; +Exposure = non-missing outcome and exposure; Final = complete case (outcome + exposure + covariates). Exclusions show the number excluded at each stage. Covariates include age at assessment, sex, maternal age, maternal education, and maternal ethnicity. BPD = Bronchopulmonary Dysplasia; IVH = Intraventricular Hemorrhage; NEC = Necrotizing Enterocolitis; HUI-3 = Health Utilities Index Mark 3; SF-6D = Short Form 6 Dimensions

**Table 25:** Variables included in the inverse probability weighting (IPW) model

Domain	Variables
Infant characteristics	Sex, gestational age, birth weight
Maternal characteristics	Maternal age, mat education, mat ethnicity, mat marital status

**Table 26:** Comparison of unweighted vs IPW-weighted regression coefficients

Exposure	Time (y)	Unweighted		IPW-weighted		$\Delta \beta$
		*β *	*P*	*β *	*P*	
BPD	19	0.001	0.976	0.023	0.489	+ 0.022
	28	− 0.029	0.499	− 0.032	0.562	− 0.003
	35	− 0.047	0.109	− 0.050	0.275	− 0.003
Severe IVH (Grade 3–4)	19	− 0.080	0.048	− 0.088	0.046	− 0.007
	28	− 0.128	0.007	− 0.146	0.032	− 0.018
	35	− 0.073	0.024	− 0.069	0.045	+ 0.005
NEC	19	0.004	0.922	0.008	0.802	+ 0.004
	28	0.027	0.567	0.034	0.433	+ 0.008
	35	− 0.045	0.135	− 0.032	0.512	+ 0.013

All models adjusted for sex, maternal age, maternal education, and maternal ethnicity. $\Delta \beta$ = IPW coefficient minus unweighted coefficient. Negative values indicate IPW estimates are larger in magnitude (more negative)

**Table 27:** Sensitivity analysis for severe IVH (Grade 3–4)

Analysis	Time (y)	*β *	95% CI	*p*-value
Best case	19	0.028	[− 0.040, 0.096]	0.425
Worst case	19	− 0.154	[− 0.215, − 0.093]	< 0.001
Best case	28	0.136	[0.073, 0.198]	<0.001
Worst case	28	− 0.256	[− 0.302, − 0.210]	< 0.001
Best case	35	0.093	[0.050, 0.136]	< 0.001
Worst case	35	− 0.170	[− 0.207, − 0.133]	< 0.001

Best case assumes missing exposed participants had excellent outcomes (90th percentile) while missing unexposed had poor outcomes (10th percentile). Worst case assumes the reverse

## Appendix 5

See Table 28.

**Table 28:** Post-hoc power analysis: MDES vs observed effect sizes

Complication	Time (y)	N (Exp)	N (Unexp)	Observed *Δ *	MDES	Power (%)
BPD	19	35	609	0.019	0.088	10.2
	28	17	297	− 0.025	0.116	7.4
	35	19	351	− 0.025	0.076	12.7
Severe IVH (Grade 3–4)	19	27	455	− 0.072	0.099	45.1
	28	17	217	− 0.094	0.111	36.0
	35	15	251	− 0.064	0.084	58.7
NEC	19	39	605	0.006	0.084	5.6
	28	21	293	0.009	0.105	5.7
	35	23	347	− 0.048	0.069	26.7

MDES, Minimum Detectable Effect Size at 80% power and *α = 0.05 *. Observed *Δ * = observed mean difference in utility scores. Power = achieved post-hoc power for the observed effect

## Appendix 6

See Table 29.

**Table 29:** Projected lifetime QALY loss and monetary valuation by discount rate

Discount rate	Lifetime QALYs lost	Value £20k	Value £30k
0% (undiscounted)	2.96	£59,239	£88,859
1.5%	2.16	£43,209	£64,813
3.5% (NICE standard)	1.51	£30,258	£45,386
5.0%	1.21	£24177	£36,266

Based on observed annual QALY decrement of 0.064 for severe IVH at age 35, projected over 45 remaining life years (to age 80)

## Appendix 7

See Fig. 2.

## References

[1] Hovi, P., Vohr, B., Ment, L. R., Doyle, L. W., McGarvey, L., Morrison, K. M., Evensen, K. A. I., van der Pal, S., Grunau, R. E., Brubakk, A. M., APIC Adults Born Preterm International Collaboration. (2016). Blood pressure in young adults born at very low birth weight: Adults born preterm international collaboration. *Hypertension,**68*(4), 880–887. 27572149 10.1161/HYPERTENSIONAHA.116.08167

[2] Doyle, L. W., Spittle, A., Anderson, P. J., & Cheong, J. L. Y. (2021). School-aged neurodevelopmental outcomes for children born extremely preterm. *Archives of Disease in Childhood,**106*(9), 834–838. 34035035 10.1136/archdischild-2021-321668

[3] Doyle, L. W., Cheong, J. L. Y., Burnett, A., Roberts, G., Lee, K. J., Anderson, P. J., Victorian Infant Collaborative Study Group (2015). Biological and social influences on outcomes of extreme-preterm/low-birth weight adolescents. *Pediatrics,**136*(6), e1513–e1520. 26553187 10.1542/peds.2015-2006

[4] Pascal, A., Govaert, P., Oostra, A., Naulaers, G., Ortibus, E., & Van den Broeck, C. (2018). Neurodevelopmental outcome in very preterm and very-low-birthweight infants born over the past decade: A meta-analytic review. *Developmental Medicine & Child Neurology,**60*(4), 342–355. 29350401 10.1111/dmcn.13675

[5] Petrou, S., Krabuanrat, N., & Khan, K. (2020). Preference-based health-related quality of life outcomes associated with preterm birth: A systematic review and meta-analysis. *Pharmacoeconomics,**38*(4), 357–373. 31814079 10.1007/s40273-019-00865-7

[6] Petrou, S., Yiu, H. H., & Kwon, J. (2019). Economic consequences of preterm birth: A systematic review of the recent literature (2009–2017). *Archives of Disease in Childhood,**104*(5), 456–465. 30413489 10.1136/archdischild-2018-315778

[7] van der Pal, S., Steinhof, M., Grevinga, M., Wolke, D., & Verrips, G. (2020). Quality of life of adults born very preterm or very low birth weight: A systematic review. *Acta Paediatrica,**109*(10), 1974–1988. 32219891 10.1111/apa.15249 PMC7891403

[8] Moster, D., Lie, R. T., & Markestad, T. (2008). Long-term medical and social consequences of preterm birth. *New England Journal of Medicine,**359*(3), 262–273. 18635431 10.1056/NEJMoa0706475

[9] Chan, P. Y. L., Morris, J. M., Leslie, G. I., Kelly, P. J., & Gallery, E. D. M. (2010). The long-term effects of prematurity and intrauterine growth restriction on cardiovascular, renal, and metabolic function. *International Journal of Pediatrics,**2010*(1), 280402. 21197428 10.1155/2010/280402 PMC3010629

[10] Serenius, F., Källén, K., Blennow, M., Ewald, U., Fellman, V., Holmström, G., Lindberg, E., Lundqvist, P., Maršál, K., Norman, M., & Olhager, E. (2013). Neurodevelopmental outcome in extremely preterm infants at 2.5 years after active perinatal care in Sweden. *JAMA,**309*(17), 1810–1820. 23632725 10.1001/jama.2013.3786

[11] Khan, K. A., Petrou, S., Dritsaki, M., Johnson, S. J., Manktelow, B., Draper, E. S., Smith, L. K., Seaton, S. E., Marlow, N., Dorling, J., & Field, D. J. (2015). Economic costs associated with moderate and late preterm birth: A prospective population-based study. *BJOG: An International Journal of Obstetrics & Gynaecology,**122*(11), 1495–1505. 26219352 10.1111/1471-0528.13515

[12] Saigal, S., & Doyle, L. W. (2008). An overview of mortality and sequelae of preterm birth from infancy to adulthood. *The Lancet,**371*(9608), 261–269. 10.1016/S0140-6736(08)60136-1 18207020

[13] Olsen, J. E., Cheong, J. L. Y., Eeles, A. L., FitzGerald, T. L., Cameron, K. L., Albesher, R. A., Anderson, P. J., Doyle, L. W., & Spittle, A. J. (2020). Early general movements are associated with developmental outcomes at 4.5–5 years. *Early Human Development,**148*, 105115. 32615517 10.1016/j.earlhumdev.2020.105115

[14] Dvir, Y., Frazier, J. A., Joseph, R. M., Mokrova, I., Moore, P. S., O’Shea, T. M., Hooper, S. R., Santos, H. P., Jr., Kuban, K., ELGAN Study Investigators (2019). Psychiatric symptoms: Prevalence, co-occurrence, and functioning among extremely low gestational age newborns at age 10 years. *Journal of Developmental & Behavioral Pediatrics,**40*(9), 725–734. 31764608 10.1097/DBP.0000000000000744 PMC6884073

[15] Kelly, M. M. (2014). Assessment of life after prematurity in 9-to 10-year-old children. *MCN: The American Journal of Maternal/Child Nursing,**39*(1), 26–32. 24076745 10.1097/NMC.0b013e3182a8de8e

[16] Varni, J. W., Seid, M., & Kurtin, P. S. (2001). Pedsql™ 4.0: Reliability and validity of the Pediatric Quality of Life Inventory™ version 4.0 generic core scales in healthy and patient populations. *Medical Care,**39*(8), 800–812. 11468499 10.1097/00005650-200108000-00006

[17] Zeitlin, J., Szamotulska, K., Drewniak, N., Mohangoo, A. D., Chalmers, J., Sakkeus, L., Irgens, L., Gatt, M., Gissler, M., Blondel, B., Euro‐Peristat Preterm Study Group. (2013). Preterm birth time trends in Europe: A study of 19 countries. *BJOG: An International Journal of Obstetrics & Gynaecology,**120*(11), 1356–1365. 23700966 10.1111/1471-0528.12281 PMC4285908

[18] Tr´eluyer, L., Nuytten, A., Guellec, I., Jarreau, P.-H., Benhammou, V., Cambonie, G., Truffert, P., Marchand-Martin, L., Ancel, P. Y., & Torchin, H. (2024). Neurodevelopment and healthcare utilisation at age 5–6 years in bronchopulmonary dysplasia: An Epipage-2 cohort study. *Archives of Disease in Childhood-Fetal and Neonatal Edition,**109*(1), 26–33. 10.1136/archdischild-2023-325376 37364896

[19] Martin, M., Smith, L., Hofheimer, J. A., McGowan, E. C., O’Shea, T. M., Pastyrnak, S., Carter, B. S., Helderman, J., Check, J., Neal, C., & Roberts, M. B. (2023). Bronchopulmonary dysplasia and neurobehavioural outcomes at birth and 2 years in infants born before 30 weeks. *Archives of Disease in Childhood-Fetal and Neonatal Edition,**108*(2), 142–148. 35999044 10.1136/archdischild-2021-323405 PMC9947192

[20] Thébaud, B., Goss, K. N., Laughon, M., Whitsett, J. A., Abman, S. H., Steinhorn, R. H., Aschner, J. L., Davis, P. G., McGrath-Morrow, S. A., Soll, R. F., & Jobe, A. H. (2019). Bronchopulmonary dysplasia. *Nature Reviews Disease primers,**5*(1), 78. 31727986 10.1038/s41572-019-0127-7 PMC6986462

[21] Gou, X., Yang, L., Pan, L., & Xiao, D. (2018). Association between bronchopulmonary dysplasia and cerebral palsy in children: A meta-analysis. *British Medical Journal Open,**8*(9), e020735. 10.1136/bmjopen-2017-020735 PMC6150141 30232102

[22] Darlow, B. A., Hutchinson, J. L., Henderson-Smart, D. J., Donoghue, D. A., Simpson, J. M., Evans, N. J., Australian, and New Zealand Neonatal Network. (2005). Prenatal risk factors for severe retinopathy of prematurity among very preterm infants of the Australian and New Zealand Neonatal Network. *Pediatrics,**115*(4), 990–996. 15805375 10.1542/peds.2004-1309

[23] O’Reilly, M., Sozo, F., & Harding, R. (2013). Impact of preterm birth and bronchopulmonary dysplasia on the developing lung: Long-term consequences for respiratory health. *Clinical and Experimental Pharmacology and Physiology,**40*(11), 765–773. 23414429 10.1111/1440-1681.12068

[24] Sriram, S., Schreiber, M. D., Msall, M. E., Kuban, K. C., Joseph, R. M., O’Shea, T. M., Allred, E. N., Leviton, A., ELGAN Study Investigators. (2018). Cognitive development and quality of life associated with bpd in 10-year-olds born preterm. *Pediatrics,**141*(6), e20172719. 29773664 10.1542/peds.2017-2719 PMC6317639

[25] Brady, J. M., Zhang, H., Kirpalani, H., & DeMauro, S. B. (2019). Living with severe bronchopulmonary dysplasia—Parental views of their child’s quality of life. *The Journal of Pediatrics,**207*, 117–122. 30404737 10.1016/j.jpeds.2018.10.001

[26] Peralta, G. P., Piatti, R., Haile, S. R., Adams, M., Bassler, D., Moeller, A., Natalucci, G., & Kriemler, S. (2023). Respiratory morbidity in preschool and school-age children born very preterm and its association with parents’ health-related quality of life and family functioning. *European Journal of Pediatrics,**182*(3), 1201–1210. 36607410 10.1007/s00431-022-04783-3 PMC9817445

[27] Shennan, A. T., Dunn, M. S., Ohlsson, A., Lennox, K., & Hoskins, E. M. (1988). Abnormal pulmonary outcomes in premature infants: Prediction from oxygen requirement in the neonatal period. *Pediatrics,**82*(4), 527–532. 3174313

[28] Patole, S. (2007). Prevention and treatment of necrotising enterocolitis in preterm neonates. *Early Human Development,**83*(10), 635–642. 17826009 10.1016/j.earlhumdev.2007.07.007

[29] Bell, M. J., Ternberg, J. L., Feigin, R. D., Keating, J. P., Marshall, R., Barton, L., & Brotherton, T. (1978). Neonatal necrotizing enterocolitis: Therapeutic decisions based upon clinical staging. *Annals of Surgery,**187*(1), 1–7. 413500 10.1097/00000658-197801000-00001 PMC1396409

[30] Papile, L.-A., Burstein, J., Burstein, R., & Koffler, H. (1978). Incidence and evolution of subependymal and intraventricular hemorrhage: A study of infants with birth weights less than 1,500 gm. *The Journal of Pediatrics,**92*(4), 529–534. 305471 10.1016/s0022-3476(78)80282-0

[31] Tréluyer, L., Chevallier, M., Jarreau, P. H., Baud, O., Benhammou, V., Gire, C., Marchand-Martin, L., Marret, S., Pierrat, V., Ancel, P. Y., & Torchin, H. (2023). Intraventricular hemorrhage in very preterm children: Mortality and neurodevelopment at age 5. *Pediatrics,**151*(4), e2022059138. 36919442 10.1542/peds.2022-059138 PMC10071431

[32] Vohr, B. R. (2022). Neurodevelopmental outcomes of premature infants with intraventricular hemorrhage across a lifespan. *Seminars in Perinatology,**46*, 151594. 35379516 10.1016/j.semperi.2022.151594

[33] Romijn, M., Dhiman, P., Finken, M. J. J., van Kaam, A. H., Katz, T. A., Rotteveel, J., Schuit, E., Collins, G. S., Onland, W., & Torchin, H. (2023). Prediction models for bronchopulmonary dysplasia in preterm infants: A systematic review and meta-analysis. *The Journal of Pediatrics,**258*, 113370. 37059387 10.1016/j.jpeds.2023.01.024

[34] Aleem, S. & Greenberg, R. G. (2023). Accurate prediction of bronchopulmonary dysplasia: Are we there yet? *The Journal of Pediatrics*, *258*. 10.1016/j.jpeds.2023.03.004 36933768

[35] Keep, R. F., Hua, Y., & Xi, G. (2012). Intracerebral haemorrhage: Mechanisms of injury and therapeutic targets. *The Lancet Neurology,**11*(8), 720–731. 22698888 10.1016/S1474-4422(12)70104-7 PMC3884550

[36] Cordonnier, C., Demchuk, A., Ziai, W., & Anderson, C. S. (2018). Intracerebral haemorrhage: Current approaches to acute management. *The Lancet,**392*(10154), 1257–1268. 10.1016/S0140-6736(18)31878-6 30319113

[37] Zhu, D.-Q., Chen, Q., Xiang, Y.-L., Zhan, C.-Y., Zhang, M.-Y., Chen, C., Zhuge, Q.-C., Chen, W.-J., Yang, X.-M., & Yang, Y.-J. (2021). Predicting intraventricular hemorrhage growth with a machine learning-based, radiomics-clinical model. *Aging (Albany NY),**13*(9), 12833. 33946042 10.18632/aging.202954 PMC8148477

[38] Chandler, J. C., & Hebra, A. (2000). Necrotizing enterocolitis in infants with very low birth weight. *Seminars in Pediatric Surgery,**9*, 63–72. 10807226 10.1016/s1055-8586(00)70018-7

[39] Llanos, A. R., Moss, M. E., Pinz`on, M. C., Dye, T., Sinkin, R. A., & Kendig, J. W. (2002). Epidemiology of neonatal necrotising enterocolitis: A population-based study. *Paediatric and Perinatal Epidemiology,**16*(4), 342–349. 12445151 10.1046/j.1365-3016.2002.00445.x

[40] Moss, R. L., Kalish, L. A., Duggan, C., Johnston, P., Brandt, M. L., Dunn, J. C. Y., Ehrenkranz, R. A., Jaksic, T., Nobuhara, K., Simpson, B. J. (2008). Clinical parameters do not adequately predict outcome in necrotizing enterocolitis: A multi-institutional study. *Journal of Perinatology,**28*(10), 665–674. 18784730 10.1038/jp.2008.119

[41] Johnson, T. J., Patel, A. L., Jegier, B. J., Engstrom, J. L., & Meier, P. P. (2013). Cost of morbidities in very low birth weight infants. *The Journal of Pediatrics,**162*(2), 243–249. 22910099 10.1016/j.jpeds.2012.07.013 PMC3584449

[42] Patel, A. L., Johnson, T. J., Engstrom, J. L., Fogg, L. F., Jegier, B. J., Bigger, H. R., & Meier, P. P. (2013). Impact of early human milk on sepsis and healthcare costs in very low birth weight infants. *Journal of Perinatology,**33*(7), 514–519. 23370606 10.1038/jp.2013.2 PMC3644388

[43] Patel, R. M., Kandefer, S., Walsh, M. C., Bell, E. F., Carlo, W. A., Laptook, A. R., Sánchez, P. J., Shankaran, S., Van Meurs, K. P., Ball, M. B., & Hale, E. C. (2015). Causes and timing of death in extremely premature infants from 2000 through 2011. *New England Journal of Medicine,**372*(4), 331–340. 25607427 10.1056/NEJMoa1403489 PMC4349362

[44] Adams, M., & Bassler, D. (2019). Practice variations and rates of late onset sepsis and necrotizing enterocolitis in very preterm born infants, a review. *Translational Pediatrics,**8*(3), 212. 31413955 10.21037/tp.2019.07.02 PMC6675686

[45] Beam, A. L., Fried, I., Palmer, N., Agniel, D., Brat, G., Fox, K., Kohane, I., Sinaiko, A., Zupancic, J. A. F., & Armstrong, J. (2020). Estimates of healthcare spending for preterm and low-birthweight infants in a commercially insured population: 2008–2016. *Journal of Perinatology,**40*(7), 1091–1099. 32103158 10.1038/s41372-020-0635-z PMC7314662

[46] Park, J., Park, S.-H., Kwon, Y.-r, Yoon, S. J., Lim, J. H., Han, J. H., Shin, J. E., Eun, H. S., Park, M. S., & Lee, S. M. (2024). Long-term outcomes of very low birth weight infants with intraventricular hemorrhage: A nationwide population study from 2011 to 2019. *World Journal of Pediatrics,**20*(7), 692–700. 38615088 10.1007/s12519-024-00799-x PMC11269332

[47] Barile, J. P., Reeve, B. B., Smith, A. W., Zack, M. M., Mitchell, S. A., Kobau, R., Cella, D. F., Luncheon, C., & Thompson, W. W. (2013). Monitoring population health for Healthy People 2020: Evaluation of the NIH PROMIS® global health, CDC Healthy Days, and satisfaction with life instruments. *Quality of Life Research,**22*(6), 1201–1211. 23404737 10.1007/s11136-012-0246-z PMC4597895

[48] Moriarty, D. G., Zack, M. M., & Kobau, R. (2003). The centers for disease control and prevention’s healthy days measures–Population tracking of perceived physical and mental health over time. *Health and Quality of Life Outcomes,**1*(1), 1–8. 14498988 10.1186/1477-7525-1-37 PMC201011

[49] Furlong, W. J., Feeny, D. H., Torrance, G. W., & Barr, R. D. (2001). The health utilities index (HUI®) system for assessing health-related quality of life in clinical studies. *Annals of Medicine,**33*(5), 375–384. 11491197 10.3109/07853890109002092

[50] Feeny, D., Furlong, W., Boyle, M., & Torrance, G. W. (1995). Multi-attribute health status classification systems. *PharmacoEconomics,**7*(6), 490–502. 10155335 10.2165/00019053-199507060-00004

[51] Brazier, J., Roberts, J., & Deverill, M. (2002). The estimation of a preference-based measure of health from the sf-36. *Journal of Health Economics,**21*(2), 271–292. 11939242 10.1016/s0167-6296(01)00130-8

[52] Framework, I. C. (1992). The mos 36-item short-form health survey (sf-36): 1. Conceptual framework and item selection. *Medical Care,**30*(6), 473–83. 1593914

[53] Bolbocean, C., Anderson, P. J., Bartmann, P., Cheong, J. L. Y., Doyle, L. W., Wolke, D., & Petrou, S. (2023). Comparative evaluation of the Health Utilities Index Mark 3 and the Short Form 6D: Evidence from an individual participant data meta-analysis of very preterm and very low birthweight adults. *Quality of Life Research*. 10.1007/s11136-023-03344-x 36705795 10.1007/s11136-023-03344-x PMC10172285

[54] Kwon, J., Bolbocean, C., Onyimadu, O., Roberts, N., & Petrou, S. (2023). Psychometric performance of generic childhood multi-attribute utility instruments in preterm and low birthweight populations: A systematic review. *Children,**10*(11), 1798. 38002889 10.3390/children10111798 PMC10670192

[55] Kwon, J., Kim, S. W., Ungar, W. J., Tsiplova, K., Madan, J., & Petrou, S. (2018). A systematic review and meta-analysis of childhood health utilities. *Medical Decision Making,**38*(3), 277–305. 28990449 10.1177/0272989X17732990

[56] Kwon, J., Kim, S. W., Ungar, W. J., Tsiplova, K., Madan, J., & Petrou, S. (2019). Patterns, trends and methodological associations in the measurement and valuation of childhood health utilities. *Quality of Life Research,**28*, 1705–1724. 30783876 10.1007/s11136-019-02121-z PMC6571090

[57] Kwon, J., Freijser, L., Huynh, E., Howell, M., Chen, G., Khan, K., Daher, S., Roberts, N., Harrison, C., Smith, S., & Devlin, N. (2022). Systematic review of conceptual, age, measurement and valuation considerations for generic multidimensional childhood patient-reported outcome measures. *Pharmacoeconomics,**40*(4), 379–431. 35072935 10.1007/s40273-021-01128-0 PMC9007803

[58] National Institute for Health and Care Excellence (NICE). Guide to the methods of technology appraisal. 2022. URL https://www.nice.org.uk/about/what-we-do/ourprogrammes/nice-guidance/chte-methods-consultation 27905712

[59] Care Excellence (2013). Guide to the methods of technology appraisal 2013 [internet]. 27905712

[60] Canadian Agency for Drugs, Technologies in Health (2006) Guidelines for the economic evaluation of health technologies: Canada.

[61] Bolbocean, C., van der Pal, S., van Buuren, S., Anderson, P. J., Bartmann, P., Baumann, N., Cheong, J. L., Darlow, B. A., Doyle, L. W., Evensen, K. A. I., & Horwood, J. (2023). Health-related quality-of-life outcomes of very preterm or very low birth weight adults: Evidence from an individual participant data meta-analysis. *Pharmacoeconomics,**41*(1), 93–105. 36287335 10.1007/s40273-022-01201-2 PMC9813180

[62] Hollanders, J. J., Van Der Pal, S. M., Van Dommelen, P., Rotteveel, J., & Finken, M. J. J. (2017). Growth pattern and final height of very preterm vs. very low birth weight infants. *Pediatric Research,**82*(2), 317–323. 28422945 10.1038/pr.2017.63

[63] Van Der Pal, S. M., Van Der Meulen, S. A., Welters, S. M., Bakker, L. A., De Groot, C. J. M., Van Kaam, A. H., & Verrips, E. (2021). Reproductive risks in 35-year-old adults born very preterm and/or with very low birth weight: An observational study. *European Journal of Pediatrics,**180*, 1219–1228. 33161502 10.1007/s00431-020-03864-5 PMC7940302

[64] Hille, E. T. M., Elbertse, L., Bennebroek Gravenhorst, J., Brand, R., Verloove-Vanhorick, S. P., Dutch POPS-19 Collaborative Study Group. (2005). Nonresponse bias in a follow-up study of 19-year-old adolescents born as preterm infants. *Pediatrics,**116*(5), e662–e666. 16263980 10.1542/peds.2005-0682

[65] Pal-de Bruin, K. M., Pal, S. M., Verloove-Vanhorick, S. P., & Walther, F. J. (2015). Profiling the preterm or VLBW born adolescent; Implications of the Dutch POPS cohort follow-up studies. *Early Human Development,**91*(2), 97–102. 25590235 10.1016/j.earlhumdev.2014.12.007

[66] Van Gendt, A. W., van der Pal, S. M., Hermes, W., Walther, F. J., van Der Pal-de Bruin, K. M., & de Groot, C. J. M. (2015). Reproductive outcomes of women and men born very preterm and/or with a very low birth weight in 1983: A longitudinal cohort study in the Netherlands. *European Journal of Pediatrics,**174*, 819–825. 25504200 10.1007/s00431-014-2470-8

[67] Torrance, G. W., Feeny, D. H., Furlong, W. J., Barr, R. D., Zhang, Y., & Wang, Q. (1996). Multiattribute utility function for a comprehensive health status classification system: Health utilities index mark 2. *Medical Care,**34*(7), 702–722. 8676608 10.1097/00005650-199607000-00004

[68] Rees, P., Dronavalli, M., Carter, B., Dickson, M., Green, C., Lawler, K., Lee, E., Uebel, H., Gale, C., & Oei, J. L. (2025). School performance of preterm-born children after intraventricular hemorrhage. *JAMA Network Open,**8*(12), e2547584–e2547584. 41379448 10.1001/jamanetworkopen.2025.47584 PMC12699354

[69] Hollebrandse, N. L., Spittle, A. J., Burnett, A. C., Anderson, P. J., Roberts, G., Doyle, L. W., & Cheong, J. L. Y. (2021). School-age outcomes following intraventricular haemorrhage in infants born extremely preterm. *Archives of Disease in Childhood-Fetal and Neonatal Edition,**106*(1), 4–8. 32732377 10.1136/archdischild-2020-318989

[70] Wang, Y., Song, J., Zhang, X., Kang, W., Li, W., Yue, Y., Zhang, S., Xu, F., Wang, X., & Zhu, C. (2022). The impact of different degrees of intraventricular hemorrhage on mortality and neurological outcomes in very preterm infants: A prospective cohort study. *Frontiers in Neurology,**13*, 853417. 35386416 10.3389/fneur.2022.853417 PMC8978798

[71] van Lunenburg, A., van der Pal, S. M., van Dommelen, P., van der Pal de Bruin, K. M., BennebroekGravenhorst, J., & Verrips, G. H. (2013). Changes in quality of life into adulthood after very preterm birth and/or very low birth weight in the Netherlands. *Health and Quality of Life Outcomes,**11*(1), 51. 23531081 10.1186/1477-7525-11-51 PMC3618000

[72] Saigal, S., Stoskopf, B., Streiner, D., Paneth, N., Pinelli, J., & Boyle, M. (2006). Growth trajectories of extremely low birth weight infants from birth to young adulthood: A longitudinal, population-based study. *Pediatric Research,**60*(6), 751–758. 17065570 10.1203/01.pdr.0000246201.93662.8e

[73] UNESCO Institute for Statistics (2012). International standard classification of education: Isced 2011. *Comparative Social Research*, 30.

[74] Oster, E. (2017). Unobservable selection and coefficient stability: Theory and evidence. *Journal of Business & Economic Statistics*. 10.1080/07350015.2016.1227711

[75] Chen, X., Zhang, W., & Li, P. (2023). Linear probability model revisited: Why it works and how it should be used in health services research. *Health Services Research,**58*(6), 1234–1245.

[76] Timoneda, J. C. (2021). Estimating group fixed effects in panel data with a binary dependent variable: How the LPM outperforms logistic regression in rare events data. *Social Science Research,**93*, 102486. 33308684 10.1016/j.ssresearch.2020.102486

[77] Gomila, R. (2021). Logistic or linear? Estimating causal effects of experimental treatments on binary outcomes using regression analysis. *Journal of Experimental Psychology: General,**150*(4), 700. 32969684 10.1037/xge0000920

[78] Hellevik, O. (2009). Linear versus logistic regression when the dependent variable is a dichotomy. *Quality & Quantity,**43*(1), 59–74.

[79] Altonji, J. G., Elder, T. E., & Taber, C. R. (2005). Selection on observed and unobserved variables: Assessing the effectiveness of Catholic schools. *Journal of Political Economy,**113*(1), 151–184.

[80] Samsa, G., Edelman, D., Rothman, M. L., Williams, G. R., Lipscomb, J., & Matchar, D. (1999). Determining clinically important differences in health status measures: A general approach with illustration to the health utilities index mark II. *PharmacoEconomics,**15*, 141–155. 10351188 10.2165/00019053-199915020-00003

[81] Drummond, M. (2001). Introducing economic and quality of life measurements into clinical studies. *Annals of Medicine,**33*(5), 344–349. 11491193 10.3109/07853890109002088

[82] Grootendorst, P., Feeny, D., & Furlong, W. (2000). Health Utilities Index Mark 3: Evidence of construct validity for stroke and arthritis in a population health survey. *Medical Care,**38*(3), 290–299. 10718354 10.1097/00005650-200003000-00006

[83] Feeny, D., Furlong, W., Torrance, G. W., Goldsmith, C. H., Zhu, Z., DePauw, S., Denton, M., & Boyle, M. (2002). Multiattribute and single-attribute utility functions for the health utilities index mark III system. *Medical Care,**40*(2), 113–128. 11802084 10.1097/00005650-200202000-00006

[84] Norman, G. R., Sloan, J. A., & Wyrwich, K. W. (2003). Interpretation of changes in health-related quality of life: The remarkable universality of half a standard deviation. *Medical Care,**41*(5), 582–592. 12719681 10.1097/01.MLR.0000062554.74615.4C

[85] Drummond, M. F., Sculpher, M. J., Claxton, K., Stoddart, G. L., & Torrance, G. W. (2015). *Methods for the economic evaluation of health care programmes*. Oxford University Press.

[86] Brazier, J., Ratcliffe, J., Saloman, J., & Tsuchiya, A. (2017). *Measuring and valuing health benefits for economic evaluation*. OXFORD university press.

[87] Noto, S., & Uemura, T. (2020). Japanese health utilities index mark 3 (HUI3): Measurement properties in a community sample. *Journal of Patient-Reported Outcomes,**4*, 1–19. 10.1186/s41687-020-0175-5 PMC6987883 31997027

[88] Rees, P., Callan, C., Chadda, K. R., Vaal, M., Diviney, J., Sabti, S., Harnden, F., Gardiner, J., Battersby, C., Gale, C., & Sutcliffe, A. (2022). Preterm brain injury and neurodevelopmental outcomes: A meta-analysis. *Pediatrics,**150*(6), e2022057442. 10.1542/peds.2022-057442 36330752 10.1542/peds.2022-057442 PMC9724175

[89] Zhou, M., Wang, S., Zhang, T., Duan, S., & Wang, H. (2024). Neurodevelopmental outcomes in preterm or low birth weight infants with germinal matrix–intraventricular hemorrhage: A meta-analysis. *Pediatric Research,**95*, 625–633. 10.1038/s41390-023-02877-8 37935882 10.1038/s41390-023-02877-8 PMC10899112

[90] Wy, P. A., Rettiganti, M., Li, J., Yap, V., Barrett, K., WhitesideMansell, L., & Casey, P. H. (2015). Impact of intraventricular hemorrhage on cognitive and behavioral outcomes at 18 years of age in low birth weight preterm infants. *Journal of Perinatology,**35*(7), 511–515. 10.1038/jp.2014.244 25654365 10.1038/jp.2014.244

[91] O’Reilly, H., Johnson, S., Ni, Y., Wolke, D., & Marlow, N. (2020). Neuropsychological outcomes at 19 years of age following extremely preterm birth. *Pediatrics,**145*(2), e20192087. 10.1542/peds.2019-2087 31924688 10.1542/peds.2019-2087

[92] Rela, A., Jary, S., Williams, C., Blair, P., Hollingworth, W., Pople, I., Whitelaw, A., Luyt, K., & Odd, D. E. (2023). Quality of life at a 10-year follow-up of children born preterm with post-hemorrhagic ventricular dilatation: A cohort study. *Neonatology,**120*(6), 690–698. 10.1159/000533355 37678198 10.1159/000533355 PMC10711773

[93] Baumann, N., Bartmann, P., & Wolke, D. (2016). Health-related quality of life into adulthood after very preterm birth. *Pediatrics*. 10.1542/peds.2015-3148 27016272 10.1542/peds.2015-3148

[94] Petrou, S., Abangma, G., Johnson, S., Wolke, D., & Marlow, N. (2009). Costs and health utilities associated with extremely preterm birth: Evidence from the Epicure study. *Value in Health,**12*(8), 1124–1134. 19659702 10.1111/j.1524-4733.2009.00580.x

[95] Ni, Y., Johnson, S., Marlow, N., & Wolke, D. (2022). Reduced health-related quality of life in children born extremely preterm in 2006 compared with 1995: The Epicure studies. *Archives of Disease in Childhood-Fetal and Neonatal Edition,**107*(4), 408–413. 34697040 10.1136/archdischild-2021-322888 PMC9209681

[96] Marlow, N., Ni, Y., Lancaster, R., Suonpera, E., Bernardi, M., Fahy, A., Larsen, J., Trickett, J., Hurst, J. R., Morris, J., & Wolke, D. (2021). No change in neurodevelopment at 11 years after extremely preterm birth. *Archives of Disease in Childhood-Fetal and Neonatal Edition,**106*(4), 418–424. 33504573 10.1136/archdischild-2020-320650

[97] Roberts, G., Burnett, A. C., Lee, K. J., Cheong, J., Wood, S. J., Anderson, P. J., Doyle, L. W., Victorian Infant Collaborative Study Group. (2013). Quality of life at age 18 years after extremely preterm birth in the post-surfactant era. *The Journal of Pediatrics,**163*(4), 1008–1013. 23885966 10.1016/j.jpeds.2013.05.048

[98] Richardson, J., Khan, M. A., Iezzi, A., & Maxwell, A. (2015). Comparing and explaining differences in the magnitude, content, and sensitivity of utilities predicted by the EQ-5D, SF-6D, HUI 3, 15D, QWB, and AQOL-8D multiattribute utility instruments. *Medical Decision Making,**35*(3), 276–291. 25159172 10.1177/0272989X14543107

[99] Albrecht, G. L., & Devlieger, P. J. (1999). The disability paradox: High quality of life against all odds. *Social Science & Medicine,**48*(8), 977–988. 10390038 10.1016/s0277-9536(98)00411-0

[100] Bonanno, G. A. (2004). Loss, trauma, and human resilience: Have we underestimated the human capacity to thrive after extremely aversive events? *American Psychologist,**59*(1), 20. 14736317 10.1037/0003-066X.59.1.20

[101] Feldman, R. (2020). What is resilience: An affiliative neuroscience approach. *World Psychiatry,**19*(2), 132–150. 32394561 10.1002/wps.20729 PMC7215067

[102] Raz, S., Piercy, J. C., Heitzer, A. M., Peters, B. N., Bapp Newman, J., DeBastos, A. K., Ofen, N., Batton, B., & Batton, D. G. (2016). Neuropsychological functioning in preterm-born twins and singletons at preschool age. *Journal of the International Neuropsychological Society,**22*(9), 865–877. 27774929 10.1017/S1355617716000758

[103] Ylijoki, M., Haataja, L., Lind, A., Ekholm, E., Lehtonen, L., PIPARI study group. (2020). Neurodevelopmental outcome of preterm twins at 5 years of age. *Pediatric Research,**87*(6), 1072–1080. 31830757 10.1038/s41390-019-0688-x

[104] Fontana, C., Schiavolin, P., Ardemani, G., Amerotti, D. A., Pesenti, N., Bonfanti, C., Boggini, T., Gangi, S., Porro, M., Squarza, C., & Giannì, M. L. (2023). To be born twin: Effects on long-term neurodevelopment of very preterm infants—A cohort study. *Frontiers in Pediatrics,**11*, 1217650. 37528875 10.3389/fped.2023.1217650 PMC10389041

[105] Achana, F., Johnson, S., Ni, Y., Marlow, N., Wolke, D., Khan, K., & Petrou, S. (2022). Economic costs and health utility values associated with extremely preterm birth: Evidence from the Epicure2 cohort study. *Paediatric and Perinatal Epidemiology,**36*(5), 696–705. 35830294 10.1111/ppe.12906 PMC9543967

[106] Hua, X., Petrou, S., Coathup, V., Carson, C., Kurinczuk, J. J., Quigley, M. A., Boyle, E., Johnson, S., Macfarlane, A., & Rivero-Arias, O. (2023). Gestational age and hospital admission costs from birth to childhood: A population-based record linkage study in England. *Archives of Disease in Childhood-Fetal and Neonatal Edition,**108*(5), 485–491. 36759168 10.1136/archdischild-2022-324763 PMC10447377

[107] Imbens, G. W., & Wooldridge, J. M. (2009). Recent developments in the econometrics of program evaluation. *Journal of Economic Literature,**47*(1), 5–86.

[108] Arnold, B. F., Khush, R. S., Ramaswamy, P., London, A. G., Rajkumar, P., Ramaprabha, P., Durairaj, N., Hubbard, A. E., Balakrishnan, K., & Colford, J. M., Jr. (2010). Causal inference methods to study nonrandomized, preexisting development interventions. *Proceedings of the National Academy of Sciences,**107*(52), 22605–22610. 10.1073/pnas.1008944107 PMC3012527 21149699

[109] Howe, L. D., Tilling, K., Galobardes, B., & Lawlor, D. A. (2013). Loss to follow-up in cohort studies: Bias in estimates of socioeconomic inequalities. *Epidemiology (Cambridge, Mass.),**24*(1), 1. 23211345 10.1097/EDE.0b013e31827623b1 PMC5102324

[110] Wolke, D., Waylen, A., Samara, M., Steer, C., Goodman, R., Ford, T., & Lamberts, K. (2009). Selective drop-out in longitudinal studies and non-biased prediction of behaviour disorders. *The British Journal of Psychiatry,**195*(3), 249–256. 19721116 10.1192/bjp.bp.108.053751 PMC2802508

[111] Walters, S. J., & Brazier, J. E. (2005). Comparison of the minimally important difference for two health state utility measures: Eq-5d and sf-6d. *Quality of Life Research,**14*(6), 1523–1532. 16110932 10.1007/s11136-004-7713-0

[112] Cheong, J. L., Anderson, P. J., Burnett, A. C., Roberts, G., Davis, N., Hickey, L., Carse, E., Doyle, L. W., Victorian Infant Collaborative Study Group. (2017). Changing neurodevelopment at 8 years in children born extremely preterm since the 1990s. *Pediatrics,**139*(6), e20164086. 28814550 10.1542/peds.2016-4086

[113] Alicia J Spittle, Kate Cameron, Lex W Doyle, Jeanie L Cheong, Victorian Infant Collaborative Study Group (2018). Motor impairment trends in extremely preterm children: 1991–2005. *Pediatrics*, 141(4). 10.1542/peds.2017-3410 29567814

[114] Larsen, J., Kochhar, P., Wolke, D., Draper, E. S., Marlow, N., & Johnson, S. (2024). Comparing behavioural outcomes in children born extremely preterm between 2006 and 1995: The EPICURE studies. *European Child & Adolescent Psychiatry,**33*(5), 1517–1528. 37430147 10.1007/s00787-023-02258-w PMC11098736

